# Characterization of 30 $^{76}$Ge enriched Broad Energy Ge detectors for GERDA Phase II

**DOI:** 10.1140/epjc/s10052-019-7353-8

**Published:** 2019-11-27

**Authors:** M. Agostini, A. M. Bakalyarov, E. Andreotti, M. Balata, I. Barabanov, L. Baudis, N. Barros, C. Bauer, E. Bellotti, S. Belogurov, G. Benato, A. Bettini, L. Bezrukov, T. Bode, D. Borowicz, V. Brudanin, R. Brugnera, D. Budjáš, A. Caldwell, C. Cattadori, A. Chernogorov, V. D’Andrea, E. V. Demidova, N. Di Marco, A. Domula, E. Doroshkevich, V. Egorov, R. Falkenstein, K. Freund, A. Gangapshev, A. Garfagnini, C. Gooch, P. Grabmayr, V. Gurentsov, K. Gusev, J. Hakenmüller, A. Hegai, M. Heisel, S. Hemmer, R. Hiller, W. Hofmann, M. Hult, L. V. Inzhechik, J. Janicskó Csáthy, J. Jochum, M. Junker, V. Kazalov, Y. Kermaïdic, T. Kihm, I. V. Kirpichnikov, A. Kirsch, A. Kish, A. Klimenko, R. Kneißl, K. T. Knöpfle, O. Kochetov, V. N. Kornoukhov, V. V. Kuzminov, M. Laubenstein, A. Lazzaro, B. Lehnert, Y. Liao, M. Lindner, I. Lippi, A. Lubashevskiy, B. Lubsandorzhiev, G. Lutter, C. Macolino, B. Majorovits, W. Maneschg, G. Marissens, M. Miloradovic, R. Mingazheva, M. Misiaszek, P. Moseev, I. Nemchenok, K. Panas, L. Pandola, K. Pelczar, A. Pullia, C. Ransom, S. Riboldi, N. Rumyantseva, C. Sada, F. Salamida, M. Salathe, C. Schmitt, B. Schneider, S. Schönert, A.-K. Schütz, O. Schulz, B. Schwingenheuer, O. Selivanenko, E. Shevchik, M. Shirchenko, H. Simgen, A. Smolnikov, L. Stanco, L. Vanhoefer, A. A. Vasenko, A. Veresnikova, K. von Sturm, V. Wagner, A. Wegmann, T. Wester, C. Wiesinger, M. Wojcik, E. Yanovich, I. Zhitnikov, S. V. Zhukov, D. Zinatulina, A. J. Zsigmond, K. Zuber, G. Zuzel, GERDA Collaboration

**Affiliations:** 1 INFN Laboratori Nazionali del Gran Sasso, LNGS, Assergi, Italy; 2 INFN Laboratori Nazionali del Gran Sasso and Università degli Studi dell’Aquila, L’Aquila, Italy; 3 INFN Laboratori Nazionali del Sud, Catania, Italy; 4 Institute of Physics, Jagiellonian University, Cracow, Poland; 5 Institut für Kern- und Teilchenphysik, Technische Universität Dresden, Dresden, Germany; 6 Joint Institute for Nuclear Research, Dubna, Russia; 7 European Commission, JRC-Geel, Geel, Belgium; 8 Max-Planck-Institut für Kernphysik, Heidelberg, Germany; 9 Dipartimento di Fisica, Università Milano Bicocca, Milan, Italy; 10 INFN Milano Bicocca, Milan, Italy; 11 Dipartimento di Fisica, Università degli Studi di Milano e INFN Milano, Milan, Italy; 12 Institute for Nuclear Research of the Russian Academy of Sciences, Moscow, Russia; 13 Institute for Theoretical and Experimental Physics, NRC “Kurchatov Institute”, Moscow, Russia; 14 National Research Centre “Kurchatov Institute”, Moscow, Russia; 15 Max-Planck-Institut für Physik, Munich, Germany; 16 Physik Department and Excellence Cluster Universe, Technische Universität München, Munich, Germany; 17 Dipartimento di Fisica e Astronomia dell’Università di Padova, Padua, Italy; 18 INFN Padova, Padua, Italy; 19 Physikalisches Institut, Eberhard Karls Universität Tübingen, Tübingen, Germany; 20 Physik Institut der Universität Zürich, Zurich, Switzerland

## Abstract

The GERmanium Detector Array (Gerda) is a low background experiment located at the Laboratori Nazionali del Gran Sasso in Italy, which searches for neutrinoless double-beta decay of $^{76}$Ge into $^{76}$Se+2e$^-$. Gerda has been conceived in two phases. Phase II, which started in December 2015, features several novelties including 30 new ^76^Ge enriched detectors. These were manufactured according to the Broad Energy Germanium (BEGe) detector design that has a better background discrimination capability and energy resolution compared to formerly widely-used types. Prior to their installation, the new BEGe detectors were mounted in vacuum cryostats and characterized in detail in the Hades underground laboratory in Belgium. This paper describes the properties and the overall performance of these detectors during operation in vacuum. The characterization campaign provided not only direct input for Gerda Phase II data collection and analyses, but also allowed to study detector phenomena, detector correlations as well as to test the accuracy of pulse shape simulation codes.

## Introduction

The search for neutrinoless double-beta (0$\nu \beta \beta$) decay of $^{76}$Ge with germanium (Ge) detectors has a 50-year-long tradition. While the former experiments that were concluded in 1967 [1], 2002 [2] and 2003 [3], exclusively used the widespread semi-coaxial detector design, the more recent Gerda [4–6] and Majorana [7] setups have intensively searched for new Ge detector designs aiming at improving the background suppression compared to the semi-coaxial type. This was partly possible due to a strong cooperation with leading Ge detector manufacturers worldwide. We selected a modified version of the point contact design [8] offered as Broad Energy Ge (BEGe) detector by the company Canberra, now part of Mirion [9]. Compared to the semi-coaxial type, the average BEGe mass is typically smaller by a factor 2-3, but its design was found to lead to an improved energy resolution and superior background rejection capability via pulse shape analysis and discrimination (PSD) of the detector signals [10, 11].

Given the performance of Gerda Phase I [12] an improvement of the sensitivity for 0$\nu \beta \beta$ decay can best be achieved by lowering the background at the *Q*-value of 2039 keV of the decay. Therefore the goal of Phase II has been to improve the background index *BI* at this energy by an order of magnitude to $10^{-3}$ cts/(keV kg year). The simple PSD method of BEGe detectors allows for a the reconstruction efficiency with a small systematic uncertainty. The most recent value for *BI* of about $6^{+4}_{-3}\cdot 10^{-4}$ cts/(keV kg year) [13, 14] results in a “background free” operation until the end of Phase II.

After a test phase based on BEGe detectors of natural isotopic composition and made from material with reduced $^{76}$Ge isotope fraction [15], 30 new BEGe diodes made from Ge with enriched $^{76}$Ge isotope fraction were produced in two batches. Prior to their installation at Gerda’s experimental site at the Laboratori Nazionali del Gran Sasso (LNGS) in Assergi, Italy, the detectors underwent extensive acceptance and characterization tests in the Hades (High Activity Disposal Experimental Site) underground laboratory in Mol, Belgium [16]. This site is located only 20 km from the detector manufacturer in Olen. It provided underground storage whenever the detectors were not processed, which was required to avoid cosmic-ray activation of the Ge material. For the detector survey, a proper infrastructure called Heroica (HADES Experimental Research Of Intrinsic Crystal Appliances) was installed [17] capable of testing several BEGe detectors at the same time, partly in an automatized scanning modus.

This paper is the extension of our first characterization paper [18] discussing the production of the first batch of enriched BEGe detectors that focused on the isotopic enrichment process, detector production, activation history and operation in vacuum as well as in the Gerda liquid argon cryostat. This was achieved by means of five BEGe detectors which had already been operated test-wise during Phase I of the experiment.

The present accompanying paper concentrates on a full description of the characterization test results obtained with the 30 new BEGe detectors during their operation in vacuum cryostats in Hades. Results already presented in [18] were revised and partly improved. Section 2 describes the main properties of the crystals used for the manufacturing of the diodes. It also provides an introduction to pulse shape simulation codes that are useful not only to optimize the Ge crystal slice cut, but also for a better understanding of different phenomena observed in this work. Section 3 is dedicated to the electrical depletion behavior of the detectors, including some peculiarities and observed parameter correlations. It also introduces a useful methodology to refine the nominal operational voltage values demonstrating the advantages for the data collection in Gerda. Section 4 describes the energy resolution of the detectors and searches for dependencies on other detector quantities. Section 5 presents the results of a high precision study of the full charge collection depths and active volumes of the new BEGe detectors. These are essential ingredients for Gerda’s exposure calculation. Section 6 examines the capability to reject $\gamma$-radiation and the possibility of fine-grained surface scans to see local effects that are partly due to the crystal lattice of Ge. Summary and conclusions are given in Sect. 7.

## Detector crystals: selection and properties

### Production of Gerda Phase II BEGe detectors

*Manufacturing:* The company Canberra Industries Inc. [19] in Oak Ridge (TN), USA (short: Canberra Oak Ridge), was selected for the Ge crystal growing process. Before that the Ge was enriched to 88 % in $^{76}$Ge at the Joint Stock Company “Production Association Electrochemical Plant” (ECP) in Zelenogorsk, Russia. Then it was purified at PPM Pure Metals in Langelsheim, Germany, reaching 35.5 kg of 6N (99.9999 %) purified Ge material (cf. [18]). Using different pullers, nine crystal ingots with a typical length of $\sim$ (18–25)  cm were grown. Out of these, 30 crystal slices were successfully cut, totaling a mass of 20.77  kg. The crystal slice cutting was optimized following two criteria: firstly, obtaining the largest possible diodes out of one ingot, and secondly producing the lowest possible amount of residual material. For a given ingot the first point was obtained by selecting the largest possible diode height while avoiding an excessive net impurity concentration gradient from top to bottom. The second criterion was achieved by considering conical tail and seed ends of the ingots as well. As a result, 21 crystal slices are cylindrical, whereas 9 are conical or even double-conical.

The company Canberra Semiconductors N.V. [20] in Olen, Belgium (short: Canberra Olen), was assigned to convert the crystal slices into working diodes following the BEGe design. The crystals were processed in two batches consisting of 7 and 23 slices each. In general, the obtained diodes conserved the overall crystal slice dimensions. Only a small mass loss was induced by the creation of the insulating groove that separates the read-out p+ electrode from the n+ contact. Only in two problematic cases the mass loss was larger (cf. Sect. 2.2). In the end, the 30 diodes amounted to a mass of 20.02  kg.

**Fig. 1:**
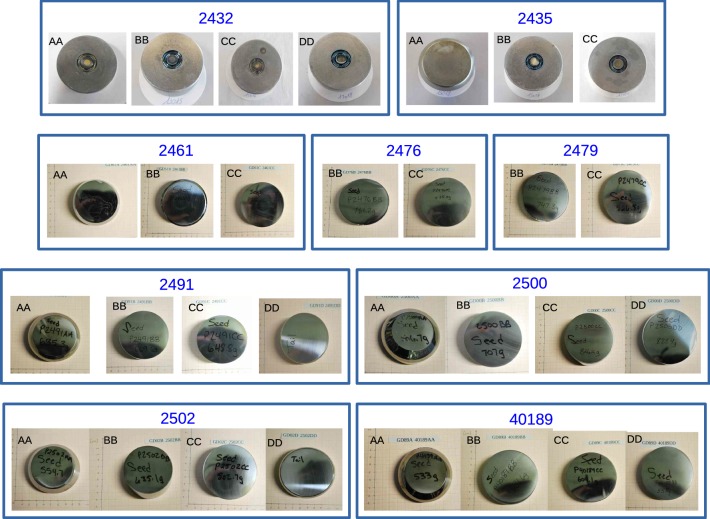
Full inventory of crystal slices/diodes belonging to the Gerda Phase II BEGe detector production. Crystals/diodes obtained from the same ingot are framed in blue. The GD32 and GD35 detector series belonging to the 1st batch are depicted in their final diode form (row 1), while the other 7 series from the 2nd batch are shown as crystal slices prior to diode conversion (rows 2–4)

*Nomenclature* The full inventory of the 30 Gerda Phase II BEGe detectors is depicted in Fig. 1. As indicated by the blue frames, 2–4 slices were obtained from one single ingot. For each slice, Canberra Oak Ridge provided a unique identifier consisting of two parts: the 4-digit serial number of the growth process with a certain puller and the relative seed- to tail-end position of a slice in terms of AA, BB, CC or DD. A few examples are 2432AA, 2476CC and 40189AA. We formed new distinct names that include both pieces of information, i.e. GD32A, GD76C and GD89A for the mentioned cases. This nomenclature is adopted in all following chapters and in all Gerda publications.

*Net impurity concentrations* The manufacturer cut thin slices at the seed- and tail-end of each single crystal ingot and measured the impurity concentrations $N_{a-d}:=$ $|N_a$-$N_d|$ via the Hall effect. Herein, $N_a$ and $N_d$ are the acceptor and donor concentrations. Further, at several axial positions of an ingot resistivity measurements were performed to determine the gradient of $N_{a-d}$ and to establish the approximate cut positions. The overall measurement precision of the $N_{a-d}$ values was quoted with ± 10 %. Within a non-disclosure agreement, we received the $N_{a-d}$ values for all crystal slices and used them for the studies presented below. In general, the $N_{a-d}$ values lie in the range [0.5,3]$\,\,\cdot \,\,10^{10}$/cm$^{3}$ (p-type material), which is ideal for high purity Ge (HPGe) detector fabrication [21]. Only in the case of GD02D, the $N_{a-d}$ value was not fully satisfactory. Therefore, the electric field strength inside this detector is expected to be deteriorated and needs special attention.

Even though the $N_{a-d}$ values vary from ingot to ingot, their absolute values typically increase from seed to tail of a single ingot. Thus, crystal slices of the same position in two ingots might differ in $N_{a-d}$, while slices of different positions in those two ingots can have very similar $N_{a-d}$ values and gradients. Nevertheless, every detector has its own impurity profile and hence electric field distribution and depletion voltage.

### Dimensions and masses of the BEGe detectors

**Fig. 2:**
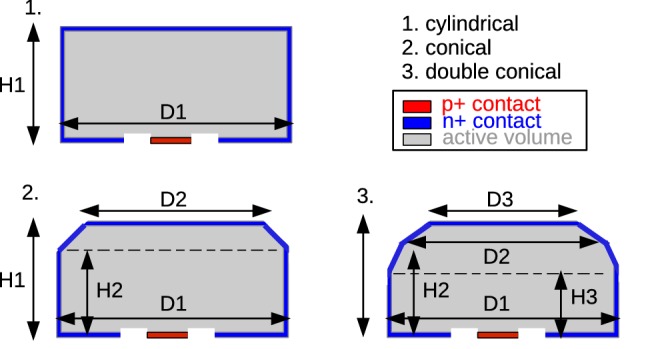
Gerda Phase II BEGe diodes are either (1) cylindrical, (2) single-conical, or (3) double-conical. Overall heights are denoted with H1, while corner heights with H2 and H3. The overall diameter is distinguished from corner diameters by D1 vs. D2, D3

*Dimensions* The dimensions of the 30 GerdaPhase II BEGe diodes were measured by Canberra Olen. In all cases, the diodes were treated as completely symmetric cylinders and accordingly only one height and one diameter per detector were quoted. Even though it could not be directly measured after diode production, the manufacturer stated, that the groove between the p$+$ and n$+$ electrodes is equal for all detectors, with an inner and outer diameter of 15 and 21  mm and a depth of $\approx$ 2.0  mm (cf. Fig. 2).

We performed a precise re-measurement, which included multiple measurements of diameters and heights at typically 4–5 different azimuth angles with respect to the *z*-axis (height) of a diode. Table 5 summarizes the mean values of the BEGe diode outer dimensions including their uncertainties as measured and used by us. The underlying terminology is explained in Fig. 2. The average diameter D1 and average height H1 of all 30 diodes are 72.8 and 29.6  mm with a standard deviation (SD) of 3.9 and 3.2  mm, respectively.

We considered detectors with conical shape separately. However, our classification distinguishes only between perfect symmetric cylindrical and conical diodes. This simplification facilitates the implementation of the individual diode geometry in Monte Carlo (MC) simulation models (cf. Sect. 5). By doing so, however, MC simulations omit a few existing facts:

- Some detectors have a slightly oval base and/or a small variation in diameter or in height. The extreme cases are detectors GD79B (diameter variation up to 0.4 %) and GD89A (height variation up to 4 %).
- Detectors GD61B, GD91D and GD32D are classified as cylindrical shaped, even though the original crystal slices had a slightly conical shape.
- Detectors GD61A, GD91A and GD00A, which are classified as conical shaped, are based on double-conical crystal slices.
- Detector GD89D, which is classified as cylindrical shaped, has a deformed shape (chopped-off edge and different heights).

All these detectors are asterisked in Table 5 and have to be treated with caution in analyses depending on the geometry of the detector volume.

*Masses* We determined the masses of the diodes with a precision of ± 1  g. The results are reported in Table 5. The average diode mass is 667  g and the SD of the detector mass distribution is 115  g. The detector mass of all 30 Gerda Phase II BEGe detectors $M=\sum _{i=1}^{30} M_i$ is (20.024 ± 0.030) kg. Herein, the $\pm 1$   g uncertainty from weighing was assumed to be correlated for all detectors. Neglecting the problematic detector GD02D (cf. Sect. 5.3), the total detector mass $M=\sum _{i=1}^{29} M_i$ is reduced to (19.362 ± 0.029)  kg.

The measurements of the single diode masses $M_m$ were also useful to compensate the geometry simplifications proposed for MC simulations. For this purpose, the analytical mass $M_a=V\cdot \rho$ was calculated using the mean dimensions and the independently determined density of the Ge crystals enriched in $^{76}$Ge, which is $\rho =5.55$  g/cm$^{3}$ [18]. Then the ratio ${\varDelta } M:=(M_a-M_m)/M_a$ was calculated. From ${\varDelta } M$ one deduces the volume difference ${\varDelta } V$ and from here a correction on the diameter and height needed to fulfill the condition ${\varDelta } M\rightarrow {\varDelta } M'\approx 0$. That way, it was possible to minimize the systematic uncertainty in MC simulations arising from the diode dimension simplification. As shown in Sect. 5, this will be of major importance for the determination of the full charge collection depths and active volumes of the detectors.

### Pulse shape simulations

The crystal slice cutting applied by the manufacturer was done in close cooperation with us. We used the net impurity concentrations $N_{a-d}$ provided by Canberra Oak Ridge and simulated the expected charge drift and signal generation on the read-out electrodes for slices of different thicknesses. The optimized cuttings were executed after feedback from our calculations. These were based on the Multi Geometry Simulation (MGS) software [22]. MGS has been also used for the prototype BEGe detector measurement campaign [23].

Within the characterization campaign of the 30 Gerda Phase II BEGe detectors, we looked for alternative field calculation and pulse shape simulation codes able to combine requirements with several advantages: easy and user-friendly adaptation of different geometries, a correct description of the field distribution inside a detector, fast processing, usage of up-to-date libraries, and the possibility to combine with Gerda related analysis software tools, i.e. the Root-based Gelatio [24] (GErda LAyouT for Input/Output) for spectral analysis, and the Geant4-based MC simulation package Mage [25] (MAjorana-GErda).

*ADL3* The Agata Detector Library ADL3 [26] is an open-source code written in the programming language C. The original code had limited field calculation possibilities, partly based on a commercial software [27], but then optimized by new algorithms and physics models [28] providing a complete pulse shape simulation framework, once the fields are calculated. The code is easily extensible and flexible enough to allow adaptation to any detector geometry and detector segmentation.

*mjd_fieldgen/mjd_siggen* The code mjd_fieldgen/mj d_siggen (short: siggen) [29–32] is an open-source code written in C. It provides an electric and weighting potential calculation and powerful pulse shape simulation for energy depositions at specific locations inside the detector. With respect to ADL3, however, it was not so flexible and required editing of the existing programs for the implementation of more complex geometries at that time.

We started with the ADL3 code and implemented the potential calculation algorithm used in siggen into ADL3 to complete the software into a full detector simulation library [30]. The following modifications were applied:

- Description of variable permittivity in a medium (important for groove simulation),
- Implementation of an electrically non-depleted region (n+ surface; later also transition layer),
- Optional 2D field calculation in cylindrical coordinates,
- Extension for optional implementation of electronic response either with or without noise.

All these features were implemented in C, so that the library can be used in the Gelatio/Mage framework. The code turned out to be very useful for diagnostics and the description of observed effects in the BEGe characterization data. Several examples are included and described in the following chapters. Besides that, the modified code has become a useful tool within and outside Gerda. For instance, it was used for the characterization and optimization of the standard BEGe detector design [30, 33], for pulse shape simulations of semi-coaxial and BEGe detectors in Gerda Phase I and II, and more recently for pulse shape studies of novel inverted semi-coaxial detectors installed in an upgrade of Gerda Phase II [34].

## Depletion and operational voltage

### Methodology: manufacturer and Gerda

*Full depletion voltage* The manufacturer Canberra Olen determines the electrical depletion voltage $V_d^C$ of a detector in a two-step approach. First, it operates the diode in a liquid nitrogen bath and measures the leakage current as well as the capacitance as a function of the applied voltage. When the capacitance reaches a ‘constant’ value, the detector is depleted by definition. This allows for a first estimation of the full depletion voltage. In a second step, Canberra Olen installs the diode in a vacuum test cryostat and irradiates it with a $\gamma$ source. The spectral positions of characteristic $\gamma$ peaks are monitored, while the voltage is increased. When the peak position of an individual $\gamma$ line stops to shift, the detector is expected to be depleted.

**Fig. 3:**
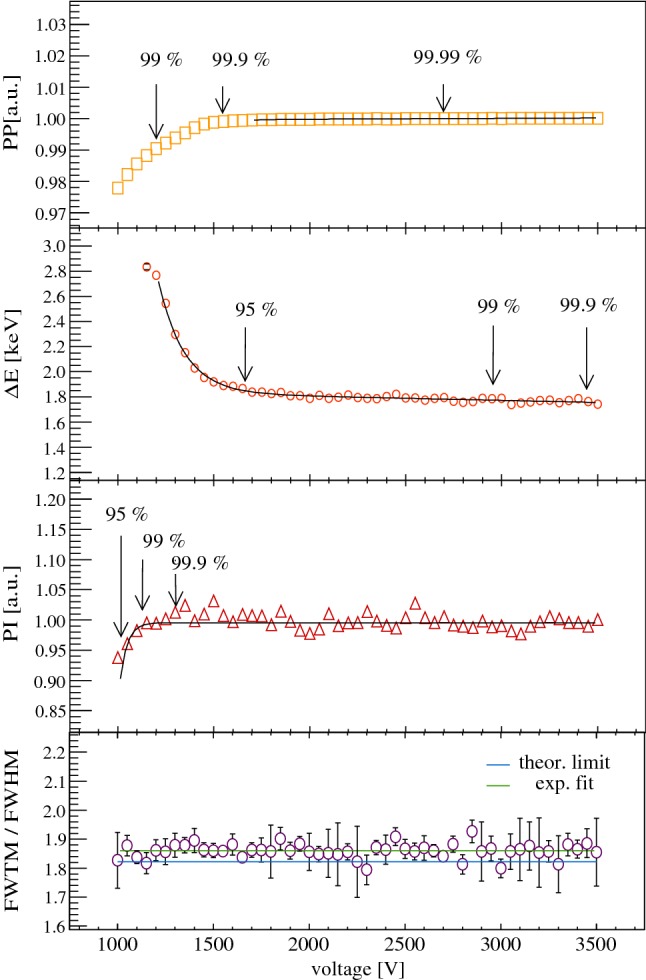
Characteristic HV scan curves of detector GD00B based on the evaluation of the 1333  keV $\gamma$ line from a $^{60}$Co calibration source. The three curves of the peak position, energy resolution and peak integral are used for the definition of the full depletion and operational voltage in Gerda. Additionally, the peak asymmetry curve in the bottom canvas demonstrates that the Gaussian peak form is conserved over a large voltage interval. More explanations are included in the text

In contrast, we use a multi-parameter approach which monitors the detector properties that are relevant for the physics goals of the experiment. The diodes installed in vacuum cryostats are irradiated with $\gamma$ sources, too. During a high voltage (HV) scan, which typically starts at 500 V and increases in 100 V steps up to the Canberra recommended voltage $V_r^C$, the following quantities of prominent $\gamma$ peaks are monitored: the peak position (*PP*), the energy resolution (${\varDelta } E$) and the peak integral (*PI*). In some cases, the peak asymmetry and pulse shape parameters are also registered. An example is shown in Fig. 3, which depicts the corresponding curves for detector GD00B. Depending on the number of peak fit parameters, the data points of the curves fluctuate more or less. Hence, the peak integral curve (depending on the correct peak shape modeling and background subtraction) typically fluctuates stronger than that for the energy resolution and peak position. In the specific case of the peak position only one parameter of a $\gamma$ line, the peak maximum, has to be extracted. The three curves are fitted with a polynomial function. Herein the plateaus encountered beyond the full depletion voltage knees are always fitted with a linear term. Based on the fit parameters, several reference depletion voltage points are extracted, at which the peak position (*PP*) reaches 99 %, 99.9 % and 99.99 % of its highest fit value obtained at $V_r^C$, the energy resolution $({\varDelta } E)$ 95 %, 99 % and 99.9 % of its smallest fit value at $V_r^C$, and the peak integral (*PI*) 95 %, 99 % and 99.9 % of its largest fit value at $V_r^C$. The corresponding voltage points are denoted with $V_d(99\,\%\,PP$) etc.

*Operational voltage* The operational voltages $V_r^C$ recommended by Canberra Olen typically lie 500–1000  V above their estimated full depletion voltages. These relatively high $V_r^C$ are still below a critical break-down voltage, but are driven by the fact that the energy resolution can still improve at the percent level within the full depletion plateau and that most customers are mainly interested in achieving the best possible energy resolution. For the Gerda experiment, however, slightly lower operational voltages might be more advantageous, for instance to keep leakage currents low or to attract less ions present in the liquid argon towards the detector surface. We defined new operational voltages $V_r^G$ which fulfill the following three criteria:

- The volume, in which a charge collection efficiency $\epsilon$ of 1 (cf. Fig. 9) can be achieved, has to be electrically fully depleted to guarantee a correct determination of the active volume and the exposure during the experimental phase. Thus, the peak integral has to be close to its maximum value and a limit of > 99 % is required.
- The energy resolution has to be close to the optimum fit value to guarantee an optimum sensitivity for the $0\nu \beta \beta$ decay of $^{76}$Ge, which scales in the presence of background as $\sqrt{(1/{\varDelta } E)}$. By default, we require > 95 % compared to the best fit value. In the realistic scenario of a 3  keV full-width at half-maximum for a peak at $Q_{\beta \beta }$($^{76}$Ge) = 2039  keV, this would correspond to a tolerable increase by 0.15  keV.
- Finally, the peak position should be stable, but does not necessarily have to be at the maximum fit value. By default, we ask for a limit better than > 99.9 %.

Typically, the following inequality holds:

1 $$\begin{aligned} V_d(99\,\% PI) < V_d(99.9\,\% PP) \end{aligned}$$

with $V_d(95\,\%\,{\varDelta } E)$ being similar to $V_d(99.9\,\%\,PP)$. The new operational voltage $V_r^G$ is defined as the voltage, which adds 500 V to the full depletion voltage $V_d$(99.9 % *PP*). This fulfills all three introduced criteria and provides enough margin to stay well above the ‘depletion knee’. The latter represents the transition region between the slopes and the plateaus of the curves.

An incomplete electronic depletion or incomplete charge collection efficiency in the Ge detector can result not only in a broader peak width, but also in the formation of peak tails to an extent that the ideal Gaussian form of a $\gamma$ peak might not be observed. A way of quantifying the effect is to measure the full-width of the peak at different heights and to calculate the related ratios $\rho$ of such widths, e.g. for the full-width at half-maximum (FWHM) and the full-width at tenth-maximum (FWTM). The ratio $\rho _{10}$ = FWTM/FWHM, which is 1.823 for a pure Gaussian peak, was calculated for voltage scans applied on individual detectors (cf. bottom of Fig. 3). In all examined cases, the experimental ratios for all voltages applied above $V_r^G$ were found to be very close to the theoretical best value. Moreover, our studies confirmed that any detector operated at a voltage above $V_r^G$ has already reached the optimum pulse shape performance (cf. Sect. 6).

### Results

*Full depletion and operational voltages* The values determined by Canberra Olen as well as by us are summarized in Table 6.

The operational voltages recommended by us are typically higher than the ones needed to reach 99 % of the optimum energy resolution, i.e. $V_d(99\,\%\,{\varDelta } E$). In all but two cases, the $V_r^G$ values are below 3.7  kV. In the case of GD91D, the applied voltage should be as high as possible. GD02D is the only detector that does not deplete completely (cf. Sects. 2.1 and 5.3). For the 29 working BEGe diodes, the average of our recommended operational voltage amounts to 3.1  kV. This is 0.6  kV lower than the average of the operational voltages recommended by Canberra Olen.

Gerda agreed to operate the new BEGe detectors at $V_r^C$. In the case of instabilities or a prohibitive increase of leakage current, however, a detector can be operated at lower voltages as long as it will not be less than $V_r^G$. In Gerda Phase II, more than four BEGe detectors have been operated at least temporarily at voltages between $V_r^G$ and $V_r^C$. Furthermore, GD02D was operated below the $V_r^G$ benchmark. More details about this problematic detector are reported in Sect. 5.3.

**Fig. 4:**
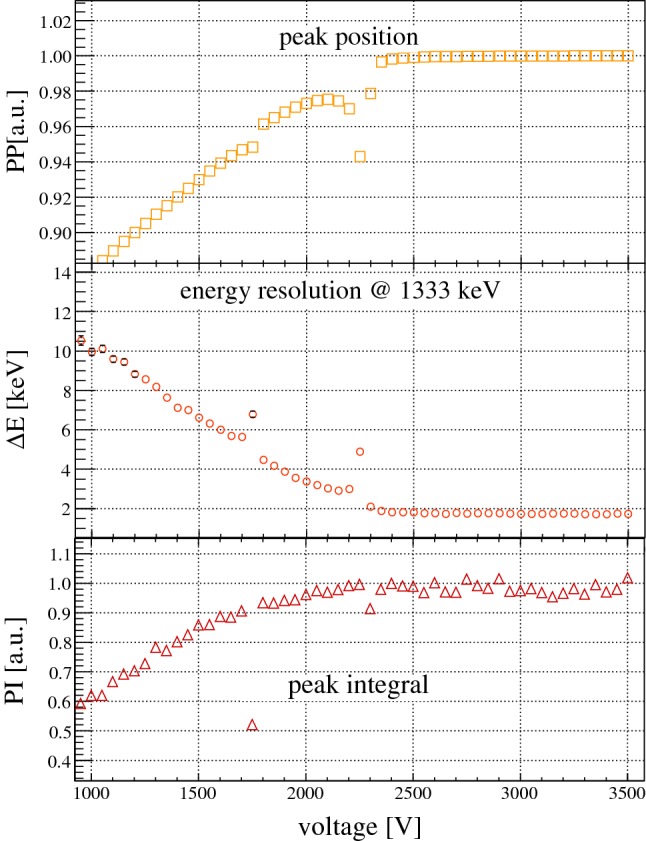
Characteristic HV scan curves of detector GD00D based on the evaluation of the 1333  keV $\gamma$ line from a $^{60}$Co calibration source. For a given curve, the distance between two values are 50 V. In all three curves of the peak position, energy resolution and peak integral the presence of two ‘bubbles’ becomes visible

*Detectors with discontinuities in the HV scans* A closer look at the normally smooth *PP* curves of the 30 Gerda Phase II BEGe detectors sometimes reveals dips that appear around the depletion voltage knees. In a few cases, the discontinuities are also observed in the corresponding ${\varDelta } E$ and in rare cases in the *PI* curves. The discontinuity behavior has been attributed to the so-called ‘bubble depletion’ [35] or ‘pinch-off’ effect [7]: For some combinations of detector geometries and net impurity concentrations the total electric field strength consisting of the applied voltage and the one from the intrinsic charge concentration can become zero in a volume around the center of the detector. This occurs for voltages just below depletion. As a consequence, the charge collection behavior changes in the sense that the holes are largely trapped or slowed down locally near the center. This leads to a reduction in the observed pulse amplitude (peak position) and potentially to a worse energy resolution and a reduction of the peak efficiency (peak count rate). Around 40 % of the Gerda Phase II BEGe detectors were found to have one or two discontinuities in the HV scans. They are listed in Table 1. Three subgroups are identified:

- Detectors with one small ‘bubble’: The weak discontinuity is seen only in the *PP* curve, but not in the energy resolution or peak integral curves. Four detectors belong to this class.
- Detectors with one large ‘bubble’: The discontinuity is clearly seen in the *PP* as well as in the ${\varDelta } E$ curve, but not in the *PI* curve. Seven detectors belong to this class.
- Detectors with two independent discontinuities: Two discontinuities at different voltages are found. They are seen in the *PP* and ${\varDelta } E$ curves, to some extent also in the *PI* curves. The discontinuity at higher voltage which is closer to the depletion knee is typically enhanced, i.e. deeper and broader. The two instabilities are separated by approximately 500 V. Two detectors belong to this class. The curves of detector GD00D are shown exemplarily in Fig. 4.

**Table 1:** List of Gerda Phase II BEGe detectors that are affected by the single or double ‘bubble depletion’ effect. Parentheses around the number of discontinuities denote a less intense effect

Detector	Discontinuities in *PP* curve	
ID	Number (#)	Voltage (kV)
GD32B	1	2.7
GD35A	1	2.4
GD35B	2	2.1; 2.7
GD61A	1	2.9
GD61C	1	2.3
GD76C	1	1.9
GD79B	(1)	2.2
GD89B	(1)	2.2
GD89D	(1)	2.3
GD91A	1	2.4
GD91B	(1)	2.5
GD00C	1	2.5
GD00D	2	1.8; 2.3

**Fig. 5:**
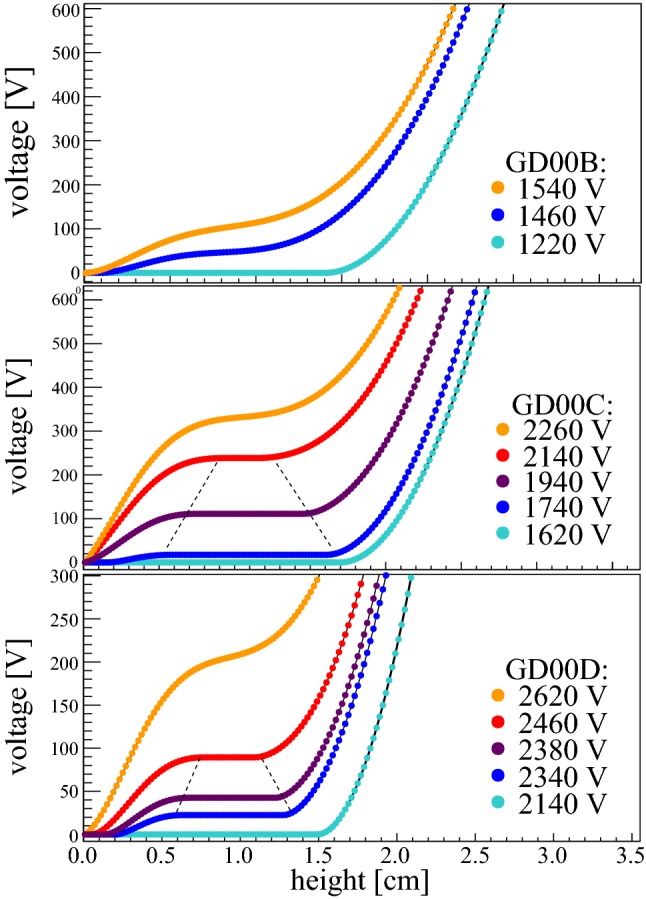
ADL3-simulation of the electrical depletion process of the detectors GD00B, GD00C and GD00D: the voltage along the central axis starting at the p+ electrode read-out is depicted as function of the voltage applied externally to the n+ contact. The curves in orange represent the voltage when the full depletion is reached. Curves with an intermediate constant interval are marked with a dotted line and correspond to voltages, at which a ‘bubble’ persists. For experimental data see Figs. 3 and 4

To our knowledge, two discontinuities at different voltages in one individual BEGe detector have been observed for the first time within this survey. In order to reproduce this scenario, we performed siggen and ADL3 simulations for detectors GD00B, GD00C and GD00D, which were produced from the same crystal ingot. Besides the exact crystal dimensions, the simulations included the net impurity concentration values provided by the manufacturer assuming a linear gradient. Figure 5 depicts exemplarily the voltage calculated with ADL3 at different heights above the read-out electrodes, i.e. along the central axes of the diodes, when different HVs are applied to the n+ electrodes. The corresponding siggen curves are not shown, but behave very similarly. In the case of detector GD00B, the simulated curves have no intermediate constant interval at or above depletion voltage, even though hardly visible in Fig. 5, and thus – like in reality – no ‘bubble’. The full depletion voltages simulated with siggen and ADL3 are in the range [1.5,1.7]  kV, which agrees well with $V_d(99.9\,\%\,PP)$. For detector GD00C the two codes predict the existence of a ‘bubble’ around [1.7,2.2]  kV and a full depletion voltage at [2.0,2.3]  kV. However, the measured ‘bubble depletion’ and full depletion voltage $V_d(99.9\,\%\,PP)$ are larger by $\sim$ 500 V. Finally, in the case of GD00D the codes predict a full depletion voltage around [2.5,2.8]  kV. This matches well with $V_d(99.9\,\%\,PP)$. But both codes foresee only one single ‘bubble’ occurring in the central bulk region when the bias voltage of [2.3,2.6]  kV is reached. The second independent discontinuity appearing at a lower voltage in the experimental data is not reproduced by the codes. A prediction of such a ‘bubble’ might require a more detailed knowledge and implementation of the radial and axial variation of the net impurity concentration. Beside this deficit, both siggen and ADL3 codes have meanwhile demonstrated to be reliable tools for the prediction of the full depletion voltage and the appearance of single ‘bubbles’ also in other HPGe detector designs (e.g. for inverted semi-coaxial Ge detectors [34]).

*Dependence of the full depletion voltage on detector parameters* This paragraph addresses the question how the full depletion voltages $V_d$ of the 29 well operating Gerda Phase II BEGe detectors depend on other detector parameters. In order to find such dependencies, one should solve the Poisson equation for the non-symmetric electric field in a BEGe detector. However, an analytical expression cannot be deduced easily and thus requires numerical calculations. On the contrary, an analytical expression for the depletion thickness *h* of a planar detector geometry can be found [21]:

2 $$\begin{aligned} h = \sqrt{\frac{2\cdot V_d \cdot \epsilon }{e\cdot N_{a-d}}} \end{aligned}$$

Here, *e* stands for the elementary electric charge and $\epsilon$ for the dielectric permittivity in the medium. The latter is defined as $\epsilon = \epsilon _0\cdot \epsilon _r$ with $\epsilon _0$ being the permittivity in vacuum and $\epsilon _r$ the relative dielectric susceptibility. In the case of Ge, $\epsilon _r$=16.0 at 295 K [36]. Since $\epsilon _r$ has only a small temperature dependence, the value is still valid for HPGe detectors operated at the boiling point of liquid argon/nitrogen at 87 K resp. 77 K [37].

Starting from Eq. (2), the following *ansatz* for the BEGe design is introduced:

3 $$\begin{aligned} k=\frac{V_d}{h^2 \cdot N_{a-d}}=\frac{e}{\epsilon }\cdot a \end{aligned}$$

with *a* being a free parameter that still has to be determined. In the case of a planar detector geometry, 1 / *a* is 2.

**Fig. 6:**
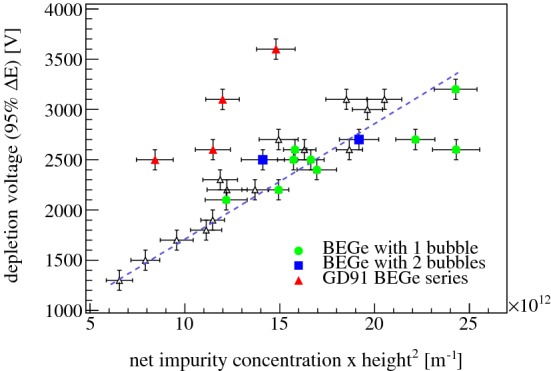
Correlation between the full depletion voltage $V_d(95\,\%$ ${\varDelta } E)$ and the product of the net impurity concentration and the squared height for all 30 Gerda Phase II BEGe detectors except for GD02D. Open symbols pertain to detectors with no appearance of the ‘bubble depletion’ effect or which do not belong to the GD91 series. The dashed line results from the fit to these 25 data points

Figure 6 depicts ${V_d}$ vs. ${h^2 \cdot N_{a-d}}$. Herein, $V_d(95\,\%{\varDelta } E)$ has been selected as ${V_d}$. The parameter $N_{a-d}$ corresponds to the net impurity concentration deduced from the crystal slice seed and tail measurements by Canberra Oak Ridge. A proportional dependence of $V_d(95\,\%{\varDelta } E)$ becomes evident, independent of the strength of $N_{a-d}$. A linear trend exists also for those situations in which the full depletion voltages defined from the *PP* and *PI* curves are used. Four detectors deviate strongly from the curve and marked in red. The detectors are GD91A, GD91B, GD91C and GD91D and belong to the same crystal ingot. A potential error of factor $\sim$ 2 in the net impurity concentration determination by the underlying Hall effect measurement would be able to explain the offset.

A linear fit of the remaining 25 points in the ${V_d}$ vs. ${h^2 \cdot N_{a-d}}$ representation has been performed. The fit parameters *k* and 1 / *a* are reported in Table 2. Contrary to a planar geometry, the 1 / *a* value for the examined BEGe detectors is close to 10 and thus $\sim$ 5 times larger.

**Table 2:** Dependence of the full depletion voltage on detector parameters following Eq. (3). The best parameters of the linear fit are reported

Detector	${V_d}$	*k*	1 / *a*
Design	[V]	[10$^{9}$ V m]	
Planar		1.13 (= *e*/(2$\epsilon$))	2
BEGe	$V_d(95\,\% {\varDelta }{E}$)	(10.5 ± 1.0)	(10.8 ± 1.0)
	$V_d(99\,\%$ PP)	(12.6 ± 1.2)	(9.0 ± 0.9)
	$V_d(95\,\%$ PI)	(10.6 ± 1.0)	(10.7 ± 1.0)

Moreover, the possibility to find detectors affected by the ‘bubble depletion’ effect in a certain region of the ${V_d}$ vs. ${h^2 \cdot N_{a-d}}$ representation was investigated. Figure 6 points towards detectors with one or two ‘bubbles’. There is no unique relation between impurity concentration and the occurrence of the ‘bubble depletion’.

## Energy resolution

### General remarks

The energy resolution ${\varDelta } E$ of a HPGe detector is defined as the width of one characteristic $\gamma$ line at a given energy *E* and consists of three sub-components:

4 $$\begin{aligned} {\varDelta } E^2 = {\varDelta } E_{sf}^2 + {\varDelta } E_{cc}^2 + {\varDelta } E_{el}^2 . \end{aligned}$$

${\varDelta } E_{sf}$ corresponds to the statistical fluctuation in the charge release and depends on the material-dependent Fano factor *F*, on the energy ${{{\mathcal {E}}}}=2.96$ eV needed for the production of one electron-hole pair in Ge at 77 K and the absorbed $\gamma$ energy *E*. ${\varDelta } E_{cc}$ corresponds to the charge carrier collection efficiency, which depends on the concentration of defects/vacancies in the bulk of the Ge crystal. It is relevant for detectors of large size and/or with low electric field strength. Finally, ${\varDelta } E_{el}$ corresponds to the electronics and environmental noise term.

In order to obtain a reproducible determination of ${\varDelta } E$ at a given $\gamma$ energy, one has to specify the measurement and analysis procedure:

- Operational detector conditions: the voltage at which ${\varDelta } E$ is measured has to be quoted. As observed in Sect. 3, the energy resolution can still improve within the depletion plateau at the level of a few percent. One has to minimize and quantify the noise contribution, e.g. via adequate pulser measurements. In our case, we further specify if the detectors are operated in cooled vacuum cryostats or bare within a cryogenic liquid.
- Energy reconstruction: the filter type (Gaussian, trapezoidal, cusp etc.) and shaping time applied for the reconstruction of the energy variable have to be defined. It should be stated whether a ballistic deficit correction, which becomes important at energies above ${\mathcal {O}}$(1 MeV) [38], is applied.
- Fit and ${\varDelta } E$ definition: the fit procedure of the $\gamma$ peak has to be specified. Within the depletion plateau, the peaks have often an almost perfect Gaussian shape. Thus, they can be fitted with a three-component function consisting of a Gaussian, a linear and a step-like term. The latter two describe the shape of the energy spectrum underlying the peak (background). The energy resolution ${\varDelta } E$ is then defined in terms of the variance $\sigma$ or the full-width at half-maximum (FWHM) of the background-subtracted Gaussian fit component of the $\gamma$ peak. If a detector has a bad crystal quality, radiation damage or cannot be fully depleted, the $\gamma$ line shape might deviate from the pure Gaussian form. The appearance of a low energy tail might be a consequence. In such cases, the fit function has to be adopted accordingly.

### Methodology: manufacturer and Gerda

The manufacturer Canberra Olen determines the energy resolution ${\varDelta } E$ of a detector in the following way: the diode is mounted in a vacuum cryostat and operated at the recommended voltage $V_r^C$. Then the detector is irradiated with non-collimated $^{57}$Co and $^{60}$Co $\gamma$ sources. The ${\varDelta } E$ of the two $\gamma$ lines at 122  keV and 1333  keV are typically expressed in terms of FWHM, whereas a potential peak distortion from the pure Gaussian shape is quantified via the measurement of the FWTM and the ratio $\rho _{10}$. The signal detection is performed with a cooled first-stage amplifying FET (20  ns rise time) as part of a front-end read-out based on the charge-sensitive Canberra 2002CSL RC-feedback preamplifier. The preamplifier has a decay constant of 47 $\upmu$s. Further an analog Canberra amplifier (e.g. Model 2022 or 2025) is used with a shaping time constant of 4 $\upmu$s. The analog-to-digital conversion (ADC) of the output signals is typically done with a standard Canberra multichannel analyzer. Finally, the manufacturer performed the spectral analysis with the Genie 2000 Gamma Analysis Software [39] following their prescribed procedures.

Within the Gerda detector characterization campaign, the ${\varDelta } E$ of the BEGe detectors operated in vacuum are evaluated mainly for the $^{60}$Co $\gamma$ line at 1333  keV, but also for other peaks originating from other sources. In general, the detectors are irradiated with non-collimated sources at a distance of typically $\sim$ 20 cm from the diode‘s top surfaces. In the case of 1333  keV, the $^{60}$Co calibration has been subdivided into:

- Standard approach: 10–60 min measurementswith a $^{60}$Co source of several 100 kBq activity are performed at the voltage $V_r^C$. ${\varDelta } E$ is extracted from this single measurement.
- Alternative approach: In order to exclude eventual temporary instabilities due to e.g. microphony from other ongoing work on-site, data collected during the HV scans described in Sect. 3 are used. The energy resolution value at $V_r^C$ is extrapolated from the polynomial fit of the ${\varDelta } E$ curve.

Signals are amplified with the same preamplifier set used by the manufacturer. Analog Canberra and ORTEC spectroscopy amplifiers were further used with an optimized shaping time constant of 8 $\upmu$s. Gerda collected data with Multi Channel Analyzer (MCA) modules by ORTEC (926,927) and Canberra (Multiport II NIM), and with Struck SIS3301 VME Flash Analog-to-Digital-Converters (FADC) [40]. The latter ones allow for a sampling-rate of 100 MHz with a 14-bit resolution per sample. Up to 128  k samples with a maximum trace length of 1.28  ms can be registered. For these energy resolution studies by Gerda the ORTEC and Canberra ADCs were used; the data were analyzed with Gelatio. The energy of an event is reconstructed with a shaping time of 8 $\upmu$s. No ballistic deficit correction is applied. The $\gamma$-peaks are fitted with the following fit function *f*(*E*):

5 $$\begin{aligned} f(E) = \frac{A}{\sqrt{2\pi }\sigma }\cdot e^{-\frac{(E-E_0)^2}{2\sigma ^2}} + \frac{B}{e^\frac{2(E-E_0)}{\sigma }+1} + C\cdot E, \end{aligned}$$

with *A*, *B*, *C* being normalizations and $\sigma$ the variance of the Gaussian distribution. The second term corresponds to a Fermi-like step function. The effect of including other step and low-side energy tail functions, as proposed in literature (see e.g. [41, 42]), was investigated for different extensions and tested on BEGe data. The impact of the fit function diversity on ${\varDelta } E$ for a fixed $V_r^C$ was estimated to be ± 0.01  keV. Only in the case of detector GD02D, the peak shape has a larger low-energy-tail even at $V_r^C$=4  kV and needs an adequate fit model extension.

### Results

*Energy resolution at 1333  keV* The energy resolutions ${\varDelta } E$ of all 30 Gerda Phase II BEGe detectors were examined according to the procedure described in Sect. 4.2. The determined values by Canberra Olen as well as by us are summarized in Table 7. The second column contains our values obtained with the method based on the HV scans. The third column shows the Gerda values obtained with the classic method based on one single measurement at $V_r^C$. The values based on the two methods sometimes disagree by $\sim$ 0.05  keV due to fit and experimental instabilities that are not considered in the total uncertainty budget. The fourth column reports the results obtained by Canberra Olen. Only in rare cases, they differ by more than $\sim$ 0.1  keV from the Gerda values. The average of all mean values quoted by Gerda and Canberra Olen are in very good agreement.

In general, the Gerda BEGe detectors have excellent energy resolutions. According to the HV scan based Gerda analysis, the average value is 1.72  keV with a SD of 0.07  keV. Further, the best detector is GD89A with (1.59 ± 0.01)  keV and the worst GD61A with (1.89 ± 0.01)  keV. Detector GD02D has an acceptable resolution of (1.84 ± 0.11)  keV, but a strong low-side energy tail due to incomplete charge collection (cf. Fig. 11).

*Dependence of energy resolution on detector parameters* This section raises the question whether the energy resolution of the 29 well working Gerda Phase II BEGe detectors is correlated to other detector parameters.

The ${\varDelta } E$ value was investigated separately for conical and cylindrical shaped detectors. No evidence was found that they would differ from each other. This further supports the decision taken during crystal production to optimize the slice cut towards a maximum mass yield. ${\varDelta } E$ turned out to be also not strongly correlated to the electronics noise term ${\varDelta } E_{el}$ in Eq. 4, which partly depends on the detector capacitance.

**Fig. 7:**
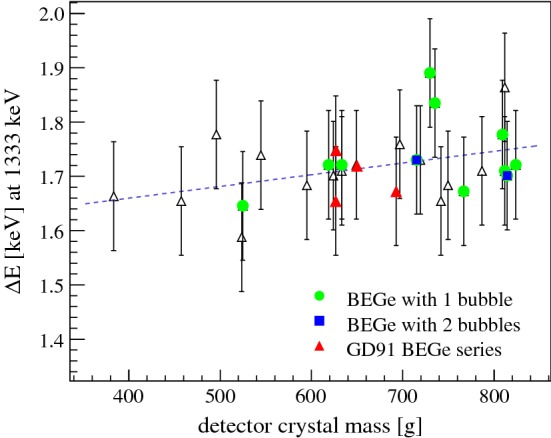
Detector energy resolution dependence on the detector mass of all 30 Gerda Phase II BEGe detectors but GD02D. Open symbols are used as in Fig. 6

Finally, ${\varDelta } E$ was plotted against the detector mass: apart from the drift times, charge collection deficits and bulk leakage currents might scale with the volume and thus with the detector mass. Figure 7 shows that a small correlation in the investigated mass range from 384  g to 824  g exists. The distribution was fitted with a linear function leading to the following relation:

6 $$\begin{aligned} {\varDelta } E(m) = 1.57(6)\,\text {keV} + m \cdot 2.2(8)\cdot 10^{-4}\,\text {keV/g} \end{aligned}$$

with *m* being the detector mass in units of gram. A dependence of the slope on the shaping time has not been investigated. Furthermore, detectors affected by the ‘bubble depletion’ effect do not appear in a clearly confined region of the parameter space.

**Fig. 8:**
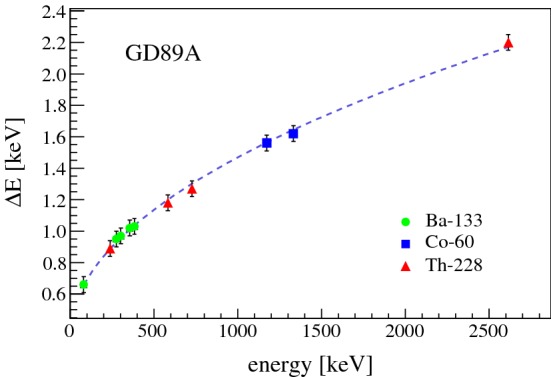
Energy resolution in terms of FWHM as function of energy for detector GD89A. For the fit function only the two energy resolution terms ${\varDelta } E_{sf}$ and ${\varDelta } E_{el}$ were considered, while ${\varDelta } E_{cc}$ was neglected

*Dependence of energy resolution on energy* For each Gerda Phase II BEGe detector $^{241}$Am, $^{133}$Ba, $^{60}$Co and $^{228}$Th calibration data were collected. This allowed to analyze for each detector the resolution for a dozen of $\gamma$ lines over energy and to deduce the energy resolution dependence from it. Figure 8 illustrates the measured ${\varDelta } E$ points for detector GD89A, which was identified to have the best energy resolution of all 30 BEGe detectors. The curve follows a $\sqrt{E}$ dependence, which arises from the charge carrier statistics term ${\varDelta } E_{sf}=2.355\cdot \sqrt{F\cdot {{{\mathcal {E}}}} \cdot E}$. This gives the opportunity to estimate the poorly known Fano factor *F*. By neglecting an expected tiny loss in energy resolution due to incomplete charge collection, one gets the following equation:

7 $$\begin{aligned} F = \frac{{\varDelta } E^2 - {\varDelta } E_{el}^2}{2.355^2\cdot {{{\mathcal {E}}}} \cdot E} \end{aligned}$$

The fit in Fig. 8 gives a noise contribution of ${\varDelta } E_{el}$=(331 ± 36) eV, which coincides well with pulser resolution measurements performed on a few BEGe detectors. The fitted value of the Fano factor is *F*=(0.079 ± 0.006). This is comparable with recently published values of *F* [43–47], which lie in the range [0.05,0.11]. For a more precise determination of *F*, a ballistic deficit correction at higher energies, a precise measurement of the noise term via an extremely stable test pulse generator, and a potential energy dependence of *F* (visible especially at lower energies) should be considered.

## Full charge collection depth and active volume

### General remarks

**Fig. 9:**
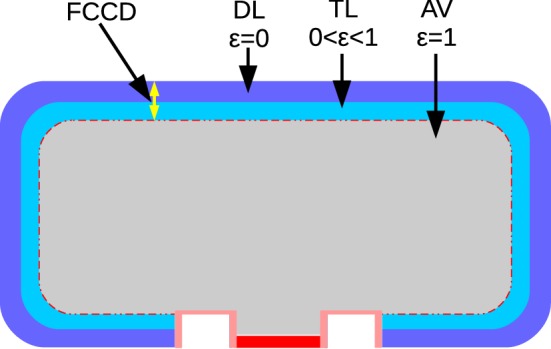
Conceptual representation of the full charge collection depth (FCCD) and active volume (AV) of the Gerda Phase II BEGe detectors. The charge collection efficiency in the dead layer (DL), transition layer (TL) and AV is denoted with $\epsilon$. Moreover, the boron (B) implanted p+ contact is depicted in red, while the inactive wrapped around lithium (Li) diffused n+ contact is drawn in blue. The insulating ring between the two electrodes is shown in pink. For further details see the text

This chapter is devoted to results for the active volume (AV) and the full charge collection depth (FCCD) of the p-type Gerda Phase II BEGe detectors. A conceptual representation of the two named quantities is depicted in Fig. 9. AV corresponds to the part of the detector volume with complete charge collection efficiency (CCE), while FCCD is a one-dimensional parameter describing the thickness of a dead layer (DL) with zero CCE plus a transition layer (TL) with partial CCE. Only particles depositing their entire energy in the AV can end up in a respective full-energy peak, which is in particular mandatory for the identification of the hypothetical 0$\nu \beta \beta$ decay. This explains why a correct determination of the AV is important for a precise exposure calculation in Gerda. Under the assumption that the FCCD is equally thick across the entire surface and there are no less efficient subregions, the AV should be equal to the crystal volume minus the volume of the surrounding layer with a thickness corresponding to the FCCD. This allows one to use either surface-sensitive low energy $\gamma$ probes to measure the FCCD directly, or bulk-sensitive high energy sources to directly probe the AV. Within this work, both types of sources have been used to deduce the FCCD and AV of the BEGe detectors. The methodology and results are presented in the following Sects. 5.2 and 5.3 respectively.

In a complementary study [48], that will not be further described here, the same calibration data were used to model the TL alone and to simulate background events that partly deposit their energy in the TL. Due to the lack of an electric field in the TL, charges have to diffuse from the TL to the AV. Since the diffusion velocity is typically smaller than the drift velocity, events generated in the TL have a longer rise time. Such characteristic ‘slow pulses’ can be efficiently rejected via pulse shape analyses techniques (cf. Sect. 6). For details see [48].

### Methodology

The basic principle behind the FCCD and AV determination is a spectral comparison of a calibration source measurement with a MC simulation, which simulates the same experimental setup and varies the FCCD around the expected one. In order to achieve the highest possible precision, several prerequisites have to be fulfilled:

- Optimized experimental setup,
- Different source types with complementary observables,
- Exact description of the experimental setup in the MC simulation,
- Careful investigation of all potential systematic effects arising from the experiment and from the MC.

In order to accomplish the first two criteria, two surface-sensitive type of sources, $^{241}$Am and $^{133}$Ba, and one bulk-sensitive source based on $^{60}$Co were selected. The $^{241}$Am and $^{133}$Ba sources typically had activities of several tens of kBq, while the $^{60}$Co sources had activities of $\sim$ (6–14) kBq. For data collection, the calibration devices were then installed at a distance of $\sim$ 20 cm from the cryostat end caps inside an optimized lead-copper shield as described in [17].

For the third criterion, the geometries of the setup, of the detectors and of the sources were implemented very accurately in the MC. The chemical composition and density of each component were investigated and re-evaluated. Especially metal components turned out to have sometimes wrong specifications. For instance, the used cryostats turned out to be made not of pure Al, but of an Al alloy with Mg, Si, Cu and Cr additions, which notably affects the absorption length of low-energy $\gamma$ rays. For the simulation part, the MC framework Mage [25] was used. The simulations included a fine-grained scan of the FCCD in 150 equidistant steps between [0,1.5]  mm.

To fulfill the fourth requisite, the impact of 34 potential systematic effects was investigated. These can originate from the MC physics processes, the radioactive sources, the properties of the cryostat and the included diode, from data collection and data analysis. A partial list containing the most relevant effects has already been reported in Table 8 of [18]. The final total uncertainty budget was divided into detector correlated and uncorrelated parts. An example for the first category is the usage of the same calibration source for each detector, which – in case of an offset – would translate into an asymmetric shift in one direction for all FCCD/AV mean values. Both terms are considered in Gerda Phase II data analyses.

**Fig. 10:**
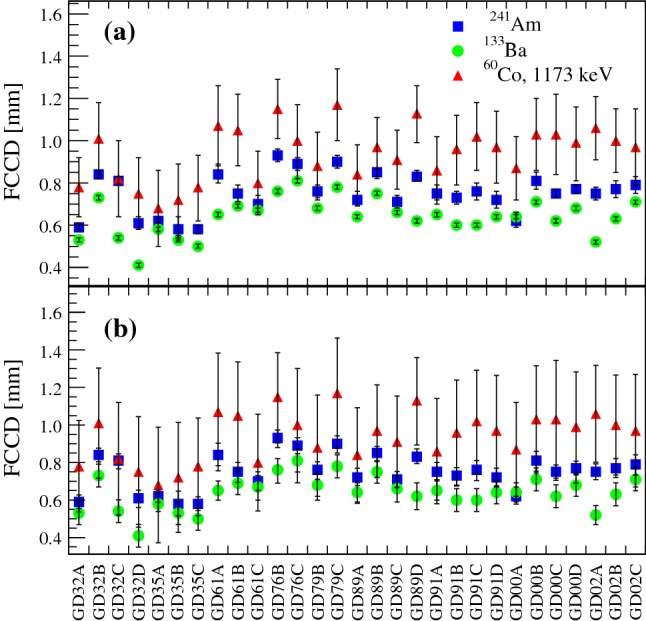
FCCD values of 29 Gerda Phase II BEGe detectors excluding GD02D. The FCCD values in plot (**a**) contain only the uncorrelated uncertainties, while in plot (**b**) they contain the combined correlated and uncorrelated uncertainties

For the analysis of the energy spectra, the $\gamma$ peaks in the measured and MC simulated spectrum were evaluated via a fitting and a counting method. In the case of the surface-sensitive measurements, two groups of $\gamma$ lines were evaluated for each source: 59.5  keV and 99.0  keV (summed with 103.0  keV) for $^{241}$Am, 79.6  keV (summed with 81.0  keV) and 356.0  keV for $^{133}$Ba. Then the peak count ratios $\rho _{exp}$ were calculated separately for each source and compared with the variable MC ratio $\rho _{mc}$. The real FCCD of the detector was established when the two ratios converge, i.e. $\rho _{exp}=\rho _{mc}$. For the bulk sensitive $^{60}$Co measurements, the absolute count rate $I_{exp}$ of one single $\gamma$ line (either 1173  keV or 1333  keV) was evaluated, whereas the source activity and dead time have to be known with high precision. Correspondingly, a MC of the same source-detector configuration was performed for variable FCCD values. The intersection of the experimental value $I_{exp}$ with the simulated curve $I_{mc}$(FCCD) is expected to agree with the real FCCD of the detector. An illustration of the two approaches can be found in Figs. 10 and 11 of [18].

### Results

*FCCD and AV from different source measurements* The FCCD results of the 29 well working Gerda Phase II BEGe detectors are reported in Table 8. The results are based on different calibration source irradiations. The first two columns summarize the FCCD values obtained from the surface sensitive $^{241}$Am and $^{133}$Ba $\gamma$ lines, while the third and fourth column represent the outcome from the two bulk sensitive $^{60}$Co $\gamma$ rays. The detectors for which the systematic effects in the determination of the FCCD were kept small are denoted with a (+) sign. The corresponding FCCD values are more reliable and can be used as reference detectors in Gerda Phase II physics data analyses. Vice versa, there are detectors with less reliable FCCD values, which are marked with (-). Detectors with e.g. an asymmetric shape and large mass difference ${\varDelta } M$ (cf. Sect. 2.2) belong to this class, since the applied mass correction might compensate the mass discrepancy, but still does not agree with the real shape. All the mentioned FCCD values are also represented in Fig. 10.

By comparing the four sets of FCCD values obtained with three different calibration sources by Gerda and with one source by Canberra Olen, it is possible to conclude:

- The $^{241}$Am-based values determined by Gerda are in good agreement with the manufacturer’s specifications. The difference is typically not larger than ± 0.1  mm. The $^{241}$Am-based average FCCD values of the first (GD32 to GD35 series) and second batch (GD61 to GD02 series) are 0.66  mm and 0.77  mm, respectively. These match well the targeted values of 0.5  mm for the first batch, and [0.7,1.0]  mm for the second.
- The FCCD values from different source measurements on a single detector agree within the combined correlated and uncorrelated uncertainties, however the mean values typically fulfill the inequality:
  8 $$\begin{aligned} \text {FCCD}(^{133}\text {Ba})<\text {FCCD}(^{241}\text {Am})<\text {FCCD}(^{60}\text {Co}) \end{aligned}$$
  This reappears in the average FCCD values of all 29 working detectors, which are summarized in Table 3. Only the two $^{60}$Co-based results agree well within uncertainties. For a BEGe detector with the average mass of 667  g, the two average FCCD values from the $^{133}$Ba and $^{60}$Co calibrations would translate into an AV fraction of 89.0 and 91.5 %, resulting in a difference of 2.5 %. None of the 34 investigated systematic effects was able to explain the discrepancy. One of few remaining, but not in depth investigated possibilities might be related to the simulated energy-dependent electron-hole cloud size induced by energy depositions in $\gamma$ source irradiations. If the Geant4 description is correct, then the observed Eq. (8) is real and would mean that the FCCD/AV is an energy-dependent quantity. However, if the description in the MC simulation was incomplete at that time, then the Eq. (8) might be due to this artifact and probably more pronounced at higher energies.

**Table 3:** Gerda Phase II BEGe detectors: averaged FCCD values based on different source types. The uncertainties include the correlated and uncorrelated averaged terms. The relative difference corresponds to the difference of $^{133}$Ba and $^{60}$Co based FCCD averaged values from the $^{241}$Am based result

Source type	Average FCCD	Rel. difference
$^{241}$Am	0.75$^{+0.04}_{-0.05}$ mm	0 %
$^{133}$Ba	0.64$^{+0.06}_{-0.06}$ mm	−15 %
$^{60}$Co, 1173 keV	0.94$^{+0.28}_{-0.28}$ mm	$+$25 %
$^{60}$Co, 1333 keV	0.95$^{+0.28}_{-0.28}$ mm	$+$27 %

By comparing the values of one specific FCCD set obtained with one source measurement, one observes a certain variation on a detector-by-detector basis. In the case of the first detector batch, the SD from the average FCCD is 0.11  mm, while it is 0.07  mm in the case of the second batch. The following effects were excluded:

- Long-term environmental conditions: The first batch was produced from February-March 2012, the second batch from August 2012 to February 2013. No seasonal correlation was found.
- Detector related properties: The detector-dependent net impurity concentrations $N_{a-d}$ seem not to influence the penetration depth of Li in Ge. Also no correlation with the slice positions was found.

Finally, no further explanations for the observed detector-by-detector variations could be found.

*GD02D, a special detector* Out of 30 delivered BEGe detectors, GD02D is the only one with a non-satisfactory net impurity concentration.

In order to characterize and quantify the electrically depleted volume, we first irradiated the detector with the bulk-sensitive $^{60}$Co source. Then the FCCD procedure was applied as previously for the well performing detectors. That way, the measured FCCD of GD02D is 7.2  mm, which translates into an AV fraction of $\sim$ 30 % only.

**Fig. 11:**
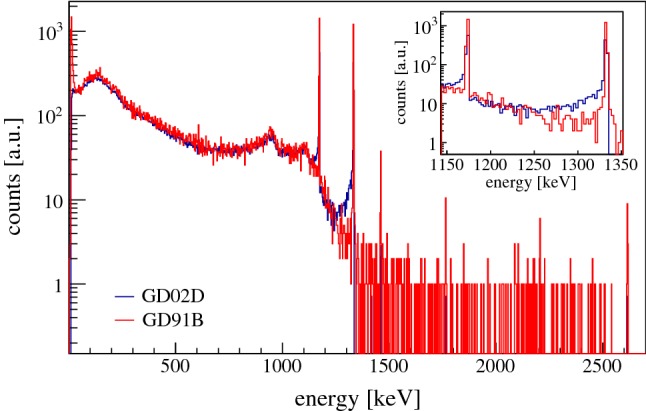
Energy spectra of the $^{60}$Co source measurements performed with the detectors GD02D and GD91B

The question was whether the residual volume is purely dead or partly also semi-active. Events depositing energy in a semi-active volume would not contribute to the full-energy peak (FEP) count rates but would be shifted to lower energies. This hypothesis was investigated by comparing GD02D with the well performing detector GD91B, since both detectors have a very similar mass, i.e. 662  g vs. 650  g, and their diameter and height are comparable: 74.6  mm vs. 70.6  mm, and 27.9  mm vs. 30.3  mm. The two detectors were calibrated with $^{60}$Co sources under almost identical conditions. The measured spectra are shown in Fig. 11. Herein, the GD02D spectrum was normalized by the measuring time of detector GD91B. No correction due to the GD02D mass surplus of 2 % and a slightly different solid angle was applied. The count rates in different ranges of the energy spectra are summarized in Table 4. The results demonstrate that the count rates in the FEPs (ID: #1 and #3) in GD02D are by factor of 2.2-2.3 lower than in GD91B. A notable part of the missing FEP events are detected in the energy intervals directly below the FEP (ID: #2 and #4). Over the entire spectrum (ID: #5 and #6), however, GD91B sees 10 % more events than GD02D. Hence, the sum of the active and semi-active volume fraction of GD02D results to be $\sim$ 80 %, out of those $\sim$ 50 % are semi-active. Thus, within Gerda Phase II the detector GD02D is used in detector-detector anti-coincidence mode only (Tables 5, 6, 7, 8).

**Table 4:** Comparison of GD02D with GD91B: count rates in different peaks and energy regions. The measurement time of GD02D was normalized to the measurement time of GD91B, and the count rates scaled accordingly

ID	Energy region	GD02D	GD91B	Factor
# 1	(1333 ± 3) keV	693	1429	2.2
# 2	[1178,1328] keV	683	406	0.6
# 3	(1173 ± 3) keV	758	1730	2.3
# 4	[1013,1168] keV	2204	2030	0.9
# 5	[100,1336] keV	34,144	38,101	1.1
# 6	[100,1000] keV	29,599	32,275	1.1

**Table 5:** Overall dimensions and masses of the 30 Gerda Phase II BEGe diodes as defined by the Gerda collaboration. These values were used for the calculation of the full charge collection depths (FCCD) and active volumes (AV) in Sect. 5. The definitions of the parameters have been introduced in Sect. 2.2. Dimensions of diodes denoted with 1(2) asterisks require (special) attention. A mismatch of detector-to-mass assignment that occurred in Table 5 of [18] for 5 detectors of the 1st batch is corrected here

Detector ID	H1	D1	H2	D2	$M_m$	${\varDelta } M$	${\varDelta } M'$
	[mm]	[mm]	[mm]	[mm]	[g]	[%]	[%]
GD32A	24.90(14)	66.26(19)	19.40(11)	60.00(18)	458	1.33	0.22
GD32B	32.16(09)	71.89(12)			716	0.74	0.13
GD32C	33.15(04)	71.99(06)			743	0.36	0.06
GD32D*	32.12(14)	72.29(19)			720	1.16	0.20
GD35A	35.34(05)	73.54(08)	22.59(03)	58.25(06)	768	0.47	0.12
GD35B	32.10(03)	76.33(04)			810	0.23	0.04
GD35C	26.32(10)	74.84(14)			634	0.87	0.09
GD61A*	33.57(10)	73.48(15)	17.44(05)	63.51(13)	731	0.91	0.21
GD61B*	30.21(09)	75.95(12)			751	0.71	0.10
GD61C	26.45(07)	74.56(10)			634	0.61	0.07
GD76B**	26.29(06)	58.27(09)			384	0.65	0.11
GD76C	33.18(07)	75.84(10)			824	0.54	0.09
GD79B*	29.04(13)	76.84(19)			736	1.1	0.14
GD79C	30.22(09)	78.95(12)			812	0.7	0.09
GD89A	28.34(08)	68.63(12)	16.34(05)	50.50(09)	524	$-$0.83	$-$0.20
GD89B	24.85(07)	76.05(09)			620	0.56	0.05
GD89C	24.75(08)	74.70(11)			595	0.67	0.06
GD89D**	22.89(28)	73.43(40)			526	2.48	0.20
GD91A*	31.18(03)	70.53(04)	19.68(02)	56.00(03)	627	$-$0.28	$-$0.07
GD91B	30.26(07)	70.58(10)			650	0.61	0.10
GD91C	29.79(08)	69.91(12)			627	0.73	0.12
GD91D*	31.88(17)	71.29(24)			693	1.44	0.25
GD00A**	26.41(15)	70.33(21)	14.35(08)	46.50(14)	496	$-$1.53	$-$0.38
GD00B	29.46(04)	73.96(06)			697	0.33	0.05
GD00C*	33.64(27)	75.52(38)			815	2.15	0.38
GD00D	32.28(07)	76.39(10)			813	0.58	0.09
GD02A	27.55(04)	70.46(06)	15.19(02)	57.50(05)	545	0.39	0.08
GD02B	28.66(04)	71.01(05)			625	0.31	0.04
GD02C	32.59(08)	74.88(11)			788	0.66	0.11
GD02D	27.91(02)	74.59(03)	21.08(02)	68.50(03)	662	$-$0.21	$-$0.03

**Table 6:** High voltage scans of Gerda Phase II BEGe detectors. Voltages *V* at which the energy resolution, the peak position and the peak integral curves reach characteristic values close to the optimum/maximum are reported. The uncertainties are around ± 200 V. The new operational voltages proposed for the usage in Gerda Phase II are denoted with $V_r^G$. The full depletion and recommended voltages $V_r^C$, which were deduced from a peak position curve by Canberra Olen, are reported in the last two columns. No data is abbreviated with ‘n.d.’

Detector ID	V [kV] by Gerda–Heroica							V [kV] by Canberra Olen	
	Best energy solution		Maximum		Highest			Highest	
			Peak integral		peak position		$V_r^G$	Peak position	$V_r^C$
	95%	99%	95%	99%	99%	99.9%		n.d.	
GD32A	2.0	2.6	1.6	2.1	1.7	2.4	2.9	2.5	3.0
GD32B	2.6	3.0	1.6	2.1	2.5	2.7	3.2	3.5	4.0
GD32C	3.1	3.7	2.5	2.9	2.9	3.2	3.7	3.5	4.0
GD32D	2.4	2.8	1.8	2.2	2.2	2.7	3.2	3.5	4.0
GD35A	2.5	2.7	2.2	2.6	2.4	2.6	3.1	3.0	4.0
GD35B	2.7	3.5	2.1	2.5	2.5	2.9	3.4	3.5	4.0
GD35C	2.7	3.3	1.9	2.3	2.1	3.0	3.5	3.0	3.5
GD61A	3.2	4.2	2.3	2.7	2.9	3.1	3.6	4.0	4.5
GD61B	3.1	3.8	2.3	2.5	2.6	3.2	3.7	3.5	4.0
GD61C	2.5	3.7	1.7	2.0	2.7	3.2	3.7	3.0	4.0
GD76B	2.2	2.5	1.6	1.8	2.0	2.7	3.2	3.0	3.5
GD76C	2.1	3.2	1.5	1.6	1.9	2.1	2.6	3.0	3.5
GD79B	2.5	2.7	1.6	1.9	2.1	2.5	3.0	3.0	3.5
GD79C	2.3	3.1	1.4	1.6	1.8	2.4	2.9	3.0	3.5
GD89A	3.0	3.7	2.1	2.3	2.5	3.1	3.6	3.5	4.0
GD89B	2.6	2.8	2.0	2.5	2.3	2.7	3.2	3.0	3.5
GD89C	2.6	3.5	2.0	2.5	2.3	2.8	3.3	3.5	4.0
GD89D	2.2	3.4	1.6	1.8	1.7	2.5	3.0	3.5	4.0
GD91A	2.5	2.7	2.0	2.7	2.2	2.6	3.1	3.0	3.5
GD91B	2.6	2.7	2.1	2.5	2.4	2.8	3.3	3.0	3.5
GD91C	3.1	3.6	2.2	2.7	2.5	3.2	3.7	3.5	4.0
GD91D	3.6	3.9	2.8	3.2	3.3	4.0	4.5	4.0	4.5
GD00A	1.3	2.1	0.8	0.9	1.0	1.2	1.7	1.5	2.5
GD00B	1.7	3.0	1.0	1.1	1.2	1.6	2.1	2.5	3.5
GD00C	2.5	2.6	2.0	2.7	2.5	2.7	3.2	3.0	3.5
GD00D	2.4	3.2	2.3	2.3	2.4	2.5	3.0	3.0	3.5
GD02A	1.5	1.6	1.0	1.1	1.3	1.7	2.2	2.0	2.5
GD02B	1.8	2.1	1.2	1.2	1.5	1.8	2.3	2.5	3.0
GD02C	2.2	2.5	1.6	1.9	1.6	2.3	2.8	2.5	3.5
GD02D	n.d.	n.d.	n.d.	n.d.	n.d.	n.d.	n.d.	3.0	4.0
Average	2.5	3.0	1.8	2.1	2.2	2.6	3.1	3.1	3.7

**Table 7:** Energy resolution of the 30 Gerda Phase II BEGe detectors at 1333  keV. All detectors were operated at the voltages $V_r^C$. The Canberra values in the 4th column were relayed without uncertainties. The Gerda-Heroica values in the 2nd and 3rd column were rounded to the relevant number of digits, i.e. two decimal places. Detectors affected by the so-called ‘bubble depletion’ effect are marked with a dagger ($\dagger$). Conical detectors are marked with an asterisk ($^{*}$). GD02D is the only detector with a remarkable low-side energy tail

Detector ID	HV scan based ${\varDelta } E$	Single meas. based ${\varDelta } E$	${\varDelta } E$
	(Gerda–Heroica)	(Gerda–Heroica)	(Canberra Olen)
	[keV]	[keV]	[keV]
GD32A$^{*}$	1.65(2)	1.71(1)	1.695
GD32B$\dagger$	1.73(1)	1.73(1)	1.747
GD32C	1.65(1)	1.65(1)	1.658
GD32D	1.73(1)	1.65(1)	1.757
GD35A$\dagger$ $^{*}$	1.67(4)	1.73(1)	1.785
GD35B$\dagger$	1.77(1)	1.77(1)	1.748
GD35C	1.71(1)	1.68(2)	1.643
GD61A$\dagger$ $^{*}$	1.89(1)	1.85(2)	1.820
GD61B	1.68(1)	1.73(2)	1.734
GD61C$\dagger$	1.72(1)	1.72(2)	1.708
GD76B	1.67(2)	1.64(3)	1.694
GD76C$\dagger$	1.72(1)	1.73(2)	1.710
GD79B$\dagger$	1.83(2)	1.86(2)	1.820
GD79C	1.87(1)	1.86(2)	1.812
GD89A$^{*}$	1.69(1)	1.68(2)	1.720
GD89B$\dagger$	1.72(3)	1.79(2)	1.684
GD89C	1.69(1)	1.75(2)	1.686
GD89D$\dagger$	1.65(1)	1.66(2)	1.721
GD91A$\dagger$ $^{*}$	1.65(3)	1.66(2)	1.746
GD91B$\dagger$	1.72(2)	1.72(2)	1.708
GD91C	1.74(2)	1.71(2)	1.708
GD91D	1.68(2)	1.68(2)	1.742
GD00A$^{*}$	1.77(1)	1.80(2)	1.724
GD00B	1.76(1)	1.84(2)	1.745
GD00C$\dagger$	1.70(1)	1.65(2)	1.762
GD00D$\dagger$	1.71(1)	1.73(2)	1.782
GD02A$^{*}$	1.74(2)	1.79(2)	1.749
GD02B	1.70(1)	1.70(3)	1.720
GD02C	1.71(1)	1.80(3)	1.748
GD02D	1.84(11)	1.73(4)	1.846

**Table 8:** FCCD results of the 29 well working Gerda Phase II BEGe detectors, as determined directly after diode production in vacuum croystats and using different probes. The spectra of the $^{241}$Am and $^{133}$Ba source measurements have been evaluated with a fitting method, while the $^{60}$Co $\gamma$ lines have been determined with a counting method. The detectors for which the systematic uncertainty was kept small are denoted with a (+) sign. Those with less reliable FCCD values have a (−) sign. The uncertainties are separated into correlated and uncorrelated components. For comparison, the $^{241}$Am-based FCCD values provided by the manufacturer are also reported. These were relayed without uncertainties

Detector ID	FCCD$^{+\text {ucorr}+\text {corr}}_{-\text {ucorr}-\text {corr}}$ by Gerda-Heroica				FCCD by Canberra
	$^{241}$Am	$^{133}$Ba	$^{60}$Co	$^{60}$Co	$^{241}$Am
	[mm]	[mm]	[mm]	[mm]	[mm]
GD32A	$0.59^{+0.03+0.02}_{-0.03-0.02}$	$0.53^{+0.06+0.01}_{-0.06-0.01}$	$0.78^{+0.20+0.14}_{-0.20-0.14}$	$0.79^{+0.19+0.14}_{-0.20-0.14}$	0.60
GD32B	$0.84^{+0.03+0.02}_{-0.03-0.02}$	$0.73^{+0.06+0.00}_{-0.06-0.01}$	$1.01^{+0.24+0.17}_{-0.24-0.17}$	$1.02^{+0.23+0.17}_{-0.24-0.17}$	0.90
GD32C$^{-}$	$0.81^{+0.03+0.02}_{-0.04-0.02}$	$0.54^{+0.06+0.01}_{-0.06-0.01}$	$0.82^{+0.24+0.18}_{-0.24-0.18}$	$0.78^{+0.23+0.17}_{-0.24-0.18}$	0.70
GD32D	$0.61^{+0.03+0.03}_{-0.04-0.03}$	$0.41^{+0.06+0.01}_{-0.06-0.01}$	$0.75^{+0.24+0.17}_{-0.24-0.17}$	$0.76^{+0.23+0.17}_{-0.24-0.17}$	0.70
GD35A$^{+}$	$0.62^{+0.03+0.01}_{-0.03-0.01}$	$0.58^{+0.05+0.01}_{-0.04-0.01}$	$0.68^{+0.25+0.18}_{-0.25-0.18}$	$0.68^{+0.24+0.18}_{-0.25-0.18}$	0.70
GD35B$^{+}$	$0.58^{+0.03+0.06}_{-0.04-0.05}$	$0.53^{+0.06+0.01}_{-0.06-0.01}$	$0.72^{+0.24+0.17}_{-0.24-0.17}$	$0.74^{+0.23+0.16}_{-0.24-0.17}$	0.70
GD35C$^{+}$	$0.58^{+0.03+0.02}_{-0.03-0.02}$	$0.50^{+0.06+0.01}_{-0.06-0.01}$	$0.78^{+0.21+0.15}_{-0.21-0.16}$	$0.73^{+0.20+0.15}_{-0.21-0.15}$	0.60
GD61A$^{-}$	$0.84^{+0.04+0.05}_{-0.04-0.04}$	$0.65^{+0.05+0.01}_{-0.05-0.01}$	$1.07^{+0.25+0.19}_{-0.25-0.19}$	$1.12^{+0.24+0.19}_{-0.25-0.19}$	0.76
GD61B	$0.75^{+0.03+0.04}_{-0.04-0.04}$	$0.69^{+0.06+0.01}_{-0.06-0.01}$	$1.05^{+0.23+0.17}_{-0.23-0.17}$	$1.06^{+0.22+0.17}_{-0.23-0.17}$	0.80
GD61C$^{+}$	$0.70^{+0.03+0.04}_{-0.03-0.04}$	$0.67^{+0.06+0.01}_{-0.07-0.01}$	$0.80^{+0.21+0.15}_{-0.21-0.15}$	$0.85^{+0.20+0.15}_{-0.21-0.15}$	0.76
GD76B$^{-}$	$0.93^{+0.03+0.03}_{-0.04-0.03}$	$0.76^{+0.06+0.01}_{-0.07-0.01}$	$1.15^{+0.19+0.14}_{-0.20-0.14}$	$1.16^{+0.19+0.14}_{-0.19-0.14}$	1.00
GD76C$^{+}$	$0.89^{+0.03+0.03}_{-0.03-0.03}$	$0.81^{+0.06+0.01}_{-0.06-0.01}$	$1.00^{+0.25+0.17}_{-0.25-0.18}$	$1.06^{+0.24+0.16}_{-0.25-0.17}$	0.92
GD79B$^{-}$	$0.76^{+0.03+0.03}_{-0.04-0.03}$	$0.68^{+0.06+0.01}_{-0.06-0.01}$	$0.88^{+0.23+0.16}_{-0.23-0.16}$	$0.88^{+0.22+0.16}_{-0.23-0.16}$	0.85
GD79C	$0.90^{+0.03+0.03}_{-0.04-0.03}$	$0.78^{+0.06+0.01}_{-0.06-0.01}$	$1.17^{+0.24+0.17}_{-0.24-0.17}$	$1.22^{+0.23+0.17}_{-0.24-0.17}$	0.90
GD89A	$0.72^{+0.03+0.04}_{-0.03-0.03}$	$0.64^{+0.05+0.01}_{-0.05-0.01}$	$0.84^{+0.21+0.14}_{-0.21-0.15}$	$0.85^{+0.21+0.15}_{-0.21-0.15}$	0.80
GD89B	$0.85^{+0.03+0.02}_{-0.04-0.02}$	$0.75^{+0.06+0.01}_{-0.06-0.01}$	$0.97^{+0.20+0.14}_{-0.20-0.15}$	$1.00^{+0.19+0.14}_{-0.20-0.15}$	0.80
GD89C$^{+}$	$0.71^{+0.03+0.03}_{-0.03-0.03}$	$0.66^{+0.06+0.01}_{-0.07-0.01}$	$0.91^{+0.20+0.14}_{-0.20-0.14}$	$0.91^{+0.19+0.14}_{-0.20-0.14}$	0.85
GD89D$^{-}$	$0.83^{+0.03+0.03}_{-0.03-0.02}$	$0.62^{+0.07+0.01}_{-0.07-0.01}$	$1.13^{+0.19+0.13}_{-0.19-0.14}$	$1.13^{+0.18+0.13}_{-0.19-0.13}$	0.76
GD91A	$0.75^{+0.03+0.04}_{-0.03-0.03}$	$0.65^{+0.05+0.01}_{-0.05-0.01}$	$0.86^{+0.23+0.16}_{-0.23-0.16}$	$0.89^{+0.22+0.16}_{-0.23-0.17}$	0.80
GD91B	$0.73^{+0.03+0.03}_{-0.04-0.03}$	$0.60^{+0.06+0.01}_{-0.06-0.01}$	$0.96^{+0.23+0.16}_{-0.23-0.17}$	$0.88^{+0.22+0.16}_{-0.23-0.17}$	0.80
GD91C	$0.76^{+0.03+0.04}_{-0.04-0.04}$	$0.60^{+0.06+0.01}_{-0.06-0.01}$	$1.02^{+0.22+0.16}_{-0.23-0.16}$	$1.03^{+0.22+0.16}_{-0.22-0.16}$	0.76
GD91D	$0.72^{+0.03+0.04}_{-0.03-0.04}$	$0.64^{+0.06+0.01}_{-0.06-0.01}$	$0.97^{+0.24+0.17}_{-0.24-0.17}$	$0.97^{+0.23+0.17}_{-0.24-0.17}$	0.80
GD00A	$0.62^{+0.03+0.04}_{-0.03-0.03}$	$0.64^{+0.05+0.01}_{-0.05-0.01}$	$0.87^{+0.20+0.15}_{-0.20-0.15}$	$0.88^{+0.19+0.15}_{-0.20-0.15}$	0.75
GD00B$^{+}$	$0.81^{+0.03+0.04}_{-0.04-0.04}$	$0.71^{+0.06+0.01}_{-0.06-0.01}$	$1.03^{+0.23+0.17}_{-0.23-0.17}$	$1.11^{+0.22+0.16}_{-0.23-0.17}$	0.76
GD00C	$0.75^{+0.03+0.02}_{-0.04-0.02}$	$0.62^{+0.06+0.01}_{-0.06-0.01}$	$1.03^{+0.25+0.19}_{-0.25-0.19}$	$1.03^{+0.24+0.19}_{-0.25-0.19}$	0.76
GD00D$^{+}$	$0.77^{+0.03+0.02}_{-0.03-0.02}$	$0.68^{+0.06+0.01}_{-0.06-0.01}$	$0.99^{+0.24+0.17}_{-0.24-0.18}$	$0.96^{+0.23+0.17}_{-0.24-0.17}$	0.80
GD02A$^{-}$	$0.75^{+0.03+0.03}_{-0.03-0.03}$	$0.52^{+0.05+0.01}_{-0.05-0.01}$	$1.06^{+0.21+0.15}_{-0.21-0.15}$	$1.05^{+0.20+0.14}_{-0.21-0.15}$	0.75
GD02B$^{+}$	$0.77^{+0.03+0.04}_{-0.03-0.04}$	$0.63^{+0.06+0.01}_{-0.06-0.01}$	$1.00^{+0.22+0.15}_{-0.22-0.15}$	$0.99^{+0.21+0.15}_{-0.22-0.15}$	0.80
GD02C$^{+}$	$0.79^{+0.03+0.04}_{-0.04-0.04}$	$0.71^{+0.06+0.01}_{-0.06-0.01}$	$0.97^{+0.24+0.18}_{-0.24-0.18}$	$0.91^{+0.24+0.18}_{-0.24-0.18}$	0.76

**Table 9:** Official dataset of FCCD and AV fractions used in Gerda Phase II physics analyses: The FCCD values were obtained by combining the $^{241}$Am FCCD and $^{133}$Ba FCCD values (1st column) and adding an offset that considers a FCCD growth at room temperature (2nd column). The offsets are individual for each detector and consider different storage periods. The AV fractions (3rd column) were deduced via subtraction of the FCCD volumes from the crystal masses. The active masses (4th column) were quoted by multiplying the crystal masses with the AV fractions

Detector ID	FCCD$^{+\text {ucorr}+\text {corr}}_{-\text {ucorr}-\text {corr}}$		$f_{av}$ $^{+\text {ucorr}+\text {corr}}_{-\text {ucorr}-\text {corr}}$	$M_{av}$ $^{+\text {ucorr}+\text {corr}}_{-\text {ucorr}-\text {corr}}$
	w/o growth	w growth	w growth	w growth
	[mm]			[g]
GD32A	0.57 $^{+ 0.03 + 0.02 }_{-0.06 - 0.02 }$	0.91 $^{+ 0.17 + 0.03 }_{- 0.17 - 0.06 }$	0.882 $^{+ 0.021 + 0.008 }_{- 0.021 - 0.004 }$	404 $^{+ 10 + 4 }_{- 10 - 2 }$
GD32B	0.80 $^{+ 0.03 + 0.02 }_{-0.06 - 0.02 }$	1.05 $^{+ 0.13 + 0.03 }_{- 0.13 - 0.06 }$	0.883 $^{+ 0.014 + 0.006 }_{- 0.014 - 0.003 }$	632 $^{+ 10 + 4 }_{- 10 - 2 }$
GD32C	0.70 $^{+ 0.03 + 0.02 }_{-0.06 - 0.02 }$	0.96 $^{+ 0.13 + 0.03 }_{- 0.13 - 0.06 }$	0.895 $^{+ 0.014 + 0.006 }_{- 0.014 - 0.003 }$	665 $^{+ 10 + 4 }_{- 10 - 2 }$
GD32D	0.52 $^{+ 0.03 + 0.03 }_{-0.06 - 0.03 }$	0.77 $^{+ 0.13 + 0.03 }_{- 0.13 - 0.06 }$	0.913 $^{+ 0.014 + 0.007 }_{- 0.014 - 0.003 }$	657 $^{+ 10 + 5 }_{- 10 - 2 }$
GD35A	0.61 $^{+ 0.03 + 0.01 }_{-0.04 - 0.01 }$	0.95 $^{+ 0.17 + 0.03 }_{- 0.17 - 0.04 }$	0.902 $^{+ 0.017 + 0.004 }_{- 0.017 - 0.003 }$	693 $^{+ 13 + 3 }_{- 13 - 2 }$
GD35B	0.55 $^{+ 0.03 + 0.06 }_{-0.06 - 0.05 }$	0.78 $^{+ 0.13 + 0.03 }_{- 0.13 - 0.06 }$	0.914 $^{+ 0.014 + 0.006 }_{- 0.014 - 0.003 }$	740 $^{+ 11 + 5 }_{- 11 - 2 }$
GD35C	0.55 $^{+ 0.03 + 0.02 }_{-0.06 - 0.02 }$	0.79 $^{+ 0.12 + 0.03 }_{- 0.12 - 0.06 }$	0.902 $^{+ 0.014 + 0.007 }_{- 0.014 - 0.004 }$	572 $^{+ 9 + 4 }_{- 9 - 3 }$
GD61A	0.72 $^{+ 0.04 + 0.05 }_{-0.05 - 0.05 }$	1.01 $^{+ 0.15 + 0.04 }_{- 0.15 - 0.05 }$	0.892 $^{+ 0.016 + 0.005 }_{- 0.015 - 0.004 }$	652 $^{+ 12 + 4 }_{- 11 - 3 }$
GD61B	0.72 $^{+ 0.03 + 0.04 }_{-0.06 - 0.04 }$	1.00 $^{+ 0.15 + 0.03 }_{- 0.15 - 0.06 }$	0.887 $^{+ 0.016 + 0.007 }_{- 0.016 - 0.003 }$	666 $^{+ 12 + 5 }_{- 12 - 2 }$
GD61C	0.68 $^{+ 0.03 + 0.04 }_{-0.07 - 0.04 }$	0.92 $^{+ 0.13 + 0.03 }_{- 0.13 - 0.07 }$	0.887 $^{+ 0.015 + 0.008 }_{- 0.015 - 0.004 }$	562 $^{+ 10 + 5 }_{- 10 - 3 }$
GD76B	0.86 $^{+ 0.03 + 0.03 }_{-0.07 - 0.03 }$	1.14 $^{+ 0.14 + 0.03 }_{- 0.14 - 0.07 }$	0.848 $^{+ 0.018 + 0.009 }_{- 0.018 - 0.004 }$	326 $^{+ 7 + 3 }_{- 7 - 2 }$
GD76C	0.85 $^{+ 0.03 + 0.03 }_{-0.06 - 0.03 }$	1.14 $^{+ 0.15 + 0.03 }_{- 0.15 - 0.06 }$	0.878 $^{+ 0.015 + 0.006 }_{- 0.015 - 0.003 }$	723 $^{+ 12 + 5 }_{- 12 - 2 }$
GD79B	0.73 $^{+ 0.03 + 0.03 }_{-0.06 - 0.03 }$	1.04 $^{+ 0.16 + 0.03 }_{- 0.16 - 0.06 }$	0.881 $^{+ 0.018 + 0.007 }_{- 0.018 - 0.003 }$	648 $^{+ 13 + 5 }_{- 13 - 2 }$
GD79C	0.85 $^{+ 0.03 + 0.03 }_{-0.06 - 0.03 }$	1.10 $^{+ 0.13 + 0.03 }_{- 0.13 - 0.06 }$	0.878 $^{+ 0.014 + 0.006 }_{- 0.014 - 0.003 }$	713 $^{+ 11 + 5 }_{- 11 - 2 }$
GD89A	0.67 $^{+ 0.03 + 0.04 }_{-0.05 - 0.03 }$	0.99 $^{+ 0.16 + 0.03 }_{- 0.16 - 0.05 }$	0.882 $^{+ 0.019 + 0.006 }_{- 0.018 - 0.003 }$	462 $^{+ 10 + 3 }_{- 9 - 2 }$
GD89B	0.82 $^{+ 0.03 + 0.02 }_{-0.06 - 0.02 }$	1.13 $^{+ 0.16 + 0.03 }_{- 0.16 - 0.06 }$	0.859 $^{+ 0.019 + 0.007 }_{- 0.019 - 0.004 }$	533 $^{+ 12 + 4 }_{- 12 - 2 }$
GD89C	0.69 $^{+ 0.03 + 0.04 }_{-0.07 - 0.04 }$	0.99 $^{+ 0.16 + 0.03 }_{- 0.16 - 0.07 }$	0.874 $^{+ 0.020 + 0.009 }_{- 0.019 - 0.004 }$	520 $^{+ 12 + 5 }_{- 11 - 2 }$
GD89D	0.75 $^{+ 0.03 + 0.03 }_{-0.07 - 0.03 }$	1.02 $^{+ 0.14 + 0.03 }_{- 0.14 - 0.07 }$	0.863 $^{+ 0.018 + 0.009 }_{- 0.018 - 0.004 }$	454 $^{+ 9 + 5 }_{- 9 - 2 }$
GD91A	0.69 $^{+ 0.03 + 0.04 }_{-0.05 - 0.03 }$	0.99 $^{+ 0.16 + 0.03 }_{- 0.15 - 0.05 }$	0.889 $^{+ 0.016 + 0.005 }_{- 0.017 - 0.003 }$	557 $^{+ 10 + 3 }_{- 11 - 2 }$
GD91B	0.68 $^{+ 0.03 + 0.03 }_{-0.06 - 0.03 }$	0.96 $^{+ 0.14 + 0.03 }_{- 0.14 - 0.06 }$	0.889 $^{+ 0.016 + 0.007 }_{- 0.016 - 0.003 }$	578 $^{+ 10 + 5 }_{- 10 - 2 }$
GD91C	0.68 $^{+ 0.03 + 0.04 }_{-0.06 - 0.04 }$	0.96 $^{+ 0.15 + 0.03 }_{- 0.15 - 0.06 }$	0.887 $^{+ 0.017 + 0.007 }_{- 0.017 - 0.003 }$	556 $^{+ 11 + 4 }_{- 11 - 2 }$
GD91D	0.68 $^{+ 0.03 + 0.04 }_{-0.06 - 0.04 }$	0.99 $^{+ 0.16 + 0.03 }_{- 0.16 - 0.06 }$	0.888 $^{+ 0.017 + 0.007 }_{- 0.017 - 0.003 }$	615 $^{+ 12 + 5 }_{- 12 - 2 }$
GD00A	0.63 $^{+ 0.03 + 0.04 }_{-0.05 - 0.03 }$	0.91 $^{+ 0.15 + 0.03 }_{- 0.14 - 0.05 }$	0.886 $^{+ 0.017 + 0.006 }_{- 0.018 - 0.004 }$	439 $^{+ 8 + 3 }_{- 9 - 2 }$
GD00B	0.76 $^{+ 0.03 + 0.04 }_{-0.06 - 0.04 }$	1.04 $^{+ 0.15 + 0.03 }_{- 0.15 - 0.06 }$	0.880 $^{+ 0.017 + 0.007 }_{- 0.017 - 0.003 }$	613 $^{+ 12 + 5 }_{- 12 - 2 }$
GD00C	0.70 $^{+ 0.03 + 0.02 }_{-0.06 - 0.02 }$	1.01 $^{+ 0.16 + 0.03 }_{- 0.16 - 0.06 }$	0.892 $^{+ 0.017 + 0.006 }_{- 0.016 - 0.003 }$	727 $^{+ 14 + 5 }_{- 13 - 2 }$
GD00D	0.73 $^{+ 0.03 + 0.02 }_{-0.06 - 0.02 }$	1.02 $^{+ 0.15 + 0.03 }_{- 0.15 - 0.06 }$	0.889 $^{+ 0.016 + 0.006 }_{- 0.016 - 0.003 }$	723 $^{+ 13 + 5 }_{- 13 - 2 }$
GD02A	0.62 $^{+ 0.03 + 0.03 }_{-0.05 - 0.03 }$	0.86 $^{+ 0.12 + 0.03 }_{- 0.12 - 0.05 }$	0.896 $^{+ 0.014 + 0.006 }_{- 0.014 - 0.003 }$	488 $^{+ 8 + 3 }_{- 8 - 2 }$
GD02B	0.70 $^{+ 0.03 + 0.04 }_{-0.06 - 0.04 }$	0.97 $^{+ 0.14 + 0.03 }_{- 0.14 - 0.06 }$	0.885 $^{+ 0.016 + 0.007 }_{- 0.016 - 0.003 }$	553 $^{+ 10 + 4 }_{- 10 - 2 }$
GD02C	0.75 $^{+ 0.03 + 0.04 }_{-0.06 - 0.04 }$	1.03 $^{+ 0.15 + 0.03 }_{- 0.15 - 0.06 }$	0.888 $^{+ 0.016 + 0.006 }_{- 0.016 - 0.003 }$	700 $^{+ 13 + 5 }_{- 13 - 2 }$

*FCCD and AV values used in GERDA Phase II* Even though the FCCD values obtained with different calibration sources agree within the total uncertainty budgets, it is important to clarify which set of FCCD mean values are more suitable for Gerda Phase II data analyses. By balancing pros and cons, we agreed to use the combined FCCD values from the independent $^{241}$Am and $^{133}$Ba source measurements (see the second column in Table 9) for the following reasons:

- The $^{241}$Am- and $^{133}$Ba-based FCCD values are determined with higher accuracy than the $^{60}$Co-based values.
- The $^{241}$Am- and $^{133}$Ba-based FCCD mean values agree better among each other than with the $^{60}$Co-based ones (cf. Table 3).
- Assuming that the hypothesis of a non-fully correct charge cloud size generation in Geant4 is true, this would mainly affect the $^{60}$Co-based values.
- Background events depositing energy in the dead and transition layer of the Li-diffused detector surfaces can be reproduced better in MC simulations, when using the $^{241}$Am- and $^{133}$Ba-based calibration data (cf. Chapter 8 in [48]).

Between the $^{241}$Am, $^{133}$Ba and $^{60}$Co source measurements of the BEGe detectors in vacuum cryostats, and the final detector deployment in Gerda in 2015, the detectors were stored at room temperature for a period of nearly 3 years. Under such conditions, the Li-diffused FCCD of HPGe detectors can increase. According to several authors [49–52] using p-type HPGe detectors from different vendors, the FCCD growth at room temperature has an average speed of $\sim$ 0.1  mm/year, with a variance of 0.04  mm/year.

Guided by these FCCD growth speed values, a correction on the combined $^{241}$Am and $^{133}$Ba FCCD and AV values was applied including a systematic uncertainty of ± 50 %. The new FCCD values, AV fractions and active masses are reported in the columns 3–5 of Table 9. The average FCCD of all BEGe detectors but GD02D increased from 0.70  mm to 0.98  mm. Its total uncertainty increased from $^{+0.04}_{-0.07}$  mm to $^{+0.15}_{-0.16}$  mm. The average AV fraction $f_{av}$ and active mass $M_{av}$ of all 29 fully operational Gerda Phase II BEGe detectors become:

9 $$\begin{aligned} f_{av}=&0.885^{+0.016}_{-0.015} \text {(uncorr)}^{+0.006}_{-0.003}\text {(corr)} \end{aligned}$$

10 $$\begin{aligned} M_{av}=&17.132^{+0.315}_{-0.294} \text {(uncorr)}^{+0.123}_{-0.063}\text {(corr)}\, \text {kg} \end{aligned}$$

Compared to the original total active mass of 17.791  kg, the new value after storage at room temperature for an averaged time of 3 yr represents an AV reduction of $\sim$ 4 %.

## Pulse shape behavior

### General remarks

The main motivation to produce detectors with a small contact area for Gerda Phase II instead of other geometries [53] is the background rejection capability via pulse shape (PS) methods. Double beta-decay signal events exhibit typically only a single localized energy deposition within a volume of a few mm$^3$ (single site event, SSE) and can therefore be discriminated against surface events and those with multiple energy depositions inside a detector (multi site event, MSE).

The BEGe pulse shape discrimination method is based on a single parameter *A* / *E*: the maximum *A* of the detector current pulse divided by the total energy *E*. The motivation and details of the implementation are discussed in our first characterization paper [18]. Another parameter of interest is the pulse rise time. Here we report on the characterization of the entire set of detectors.

### Methodology

Fine-grained surface scans with low-energy $\gamma$-ray $^{241}$Am sources were performed on most Gerda Phase II BEGe detectors to study the rise time behavior of generated pulses. Such scans were based on collimated 5 MBq $^{241}$Am sources (60  keV $\gamma$ emitters) mounted on automatized robotic scanning arms [17] and consisted typically of:

- Circular scans of the top diode surface: these were composed of up to 230 positions distributed over maximal 11 rings with radii of <35  mm. In each position $\sim$ 10$^3$ events were collected.
- Side surface scans: these comprised up to 380 positions distributed over a maximum of 11 rings at different heights. Again, $\sim$ 10$^3$ events per position were registered.

The pulse shape information from these surface scan data was used for the calculation of the mean rise time $\tau _r$ of the *Q*(*t*) pulses for different time intervals of the rising edge. By comparing the local difference in $\tau _r$, it was possible to study hole mobilities in our detectors.

To study the background rejection capability of the Gerda Phase II detectors via PS analysis, the suppression of $\gamma$-radiation was quantified. The impact of $\alpha$- and $\beta$-emitting sources on the PS could not be studied, since the Al cryostats block these particles from reaching the detector. The detectors were irradiated with non-collimated $^{228}$Th sources of (1–15)  kBq activity placed in 20 cm distance from the cryostat end caps for (8-18) hours. $^{228}$Th is advantageous, since the double escape peak (DEP) events of 2615  keV $\gamma$ rays are SSEs, while full-energy peaks (FEP) at 1621  keV and 2615  keV, the single escape peak (SEP) at 2105  keV, and Compton continua contain mostly MSEs. The *A* / *E* ratio distribution from the DEP (cf. Fig. 13 in [18]) was fitted to define a cut on *A* / *E* which keeps 90 % of the signal-like DEP events. This measure effectively excludes many background events (MSEs and ‘slow pulses’) with lower *A* / *E*, while keeping a large fraction of the SSEs.

### Results

*Rise time and anisotropic hole mobility* For every scanned position on each detector surface, the mean rise time $\tau _r$ of pulses induced by the 60  keV $\gamma$ rays was calculated. Among other possibilities, the [2,70] % and [1,90] % time intervals on the emerging pulses were favored to define the value of $\tau _r$. The obtained $\tau _r$ values determined at different scan positions were plotted as a function of either radius and angle for top surface scans, or of height and angle for side surface scans.

**Fig. 12:**
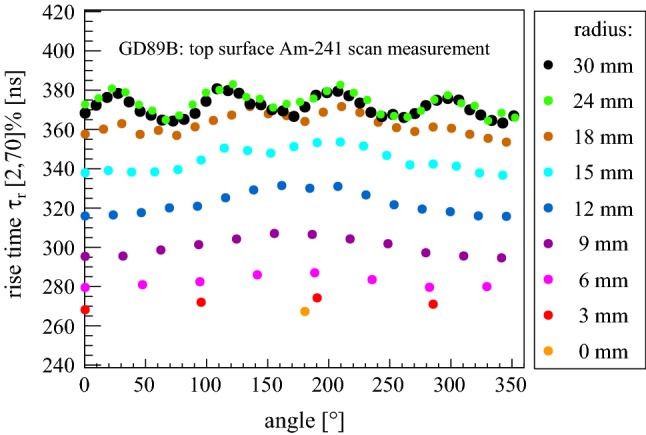
Detector GD89B: measured rise time curves of circular scans on the top diode surface, with a $\tau _r$ definition in the [2,70] % interval

The $\tau _r$([2,70] %) evaluation of the top surface scan performed on detector GD89B is depicted exemplarily in Fig. 12. At the largest radius, i.e. 30  mm, a 90$^\circ$ oscillation pattern becomes clearly visible. The $\tau _r$ has an average of $\sim$ 370  ns, while the difference between the minima and maxima is $\sim$ 20  ns, i.e. 5 %. The observed 90$^\circ$ oscillation is due to different hole drift mobilities, which depend not only on the applied electric field and the doping levels, but also along what crystallographic axis of the diamond lattice the charges drift. In case of holes, the velocity is largest along $\langle {100}\rangle$ and slowest along $\langle {111}\rangle$ [54].

The entire Gerda Phase II BEGe detector survey allowed to draw several conclusions about the observed ‘anisotropic mobility’:

- Collimated $^{241}$Am source data from top diode surface scans (especially with radii > 25  mm) are always suitable for the observation of the 90$^\circ$ oscillation.
- The $\tau _r$([2,70] %) definition is more suitable to extract the 90$^\circ$ oscillation, while the $\tau _r$([1,90] %) values are closer to the charge collection process time. The difference between the minimum and maximum rise time due to the oscillation pattern is typically $\sim$ 20  ns. The average $\tau _r$([1,90] %) value for a top scan radius of [25,30]  mm is (520 ± 50)  ns, attributed to the same read-out electrode geometry and similar outer dimensions of the detectors.
- At large radii in top surface scans, some detectors were found to have a second 180$^\circ$ oscillation that superimposes the 90$^\circ$ one (cf. Fig. 16 in [18]). It turned out that these detectors are also affected by the ‘*A* / *E* ratio anomaly’ in $^{228}$Th source data, as described in the following paragraphs. Moreover, problematic cases like GD02D have an irregular trend in all rise time curves.

**Fig. 13:**
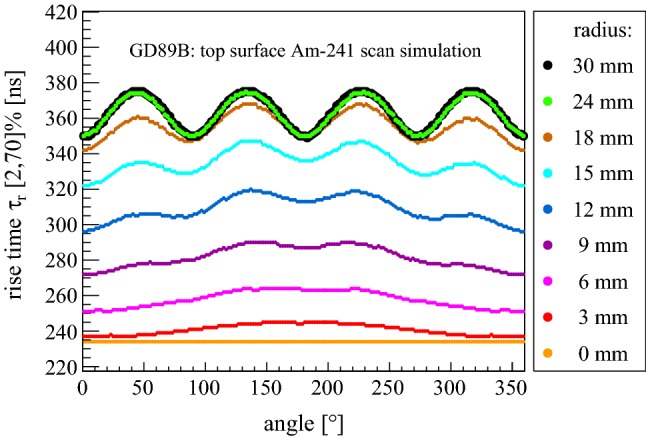
Detector GD89B: ADL3-based simulation of rise time curves of circular scans on the top diode surface using the $\tau _r$([2,70] %) definition

**Fig. 14:**
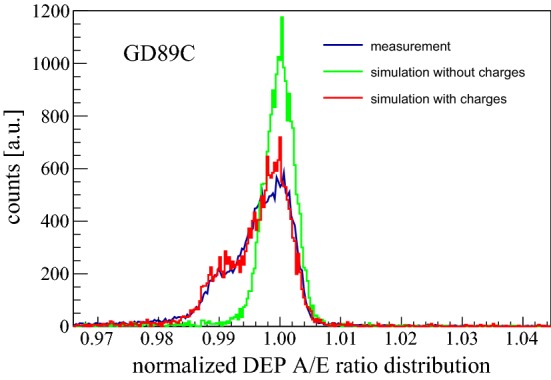
Detector GD89C: measurement vs. siggen-based simulation of the *A* / *E* ratio distribution of DEP events. In both cases, the $^{228}$Th source placed in 20 cm distance from the detector was not collimated

We performed ADL3-based simulations of the expected rise time from $^{241}$Am source scans on several BEGe detectors. The results for the top surface scan of GD89B are depicted in Fig. 13. The simulation predicts a gradual increase in rise/drift time and the 90$^\circ$ oscillation with increasing radius. The calculated $\tau _r$([2,70] %) values match the experimental data well. Moreover, at small radii of top surface scans, some detectors were found to have a modulation of the normally rather flat rise/drift time. The simulation is able to reproduce such an artifact, if a misalignment of 1  mm between the detector axis and the center of the scanning circle is assumed. This corresponds indeed to the achievable precision in the experimental alignment procedure (cf. [17]).

**Table 10:** Gamma-ray background survival fractions and the DEP *A* / *E* resolution $b_{A/E}$($^{228}$Th) for the 30 Gerda Phase II BEGe detectors operated under vacuum conditions. In the case of the detector GD32D, the pulse shape performance was measured before (I) and after (II) reprocessing. The uncertainty of the survival fraction includes the statistical and the systematic uncertainties [55]

Detector ID	DEP	SEP	FEP	FEP	ROI	$b_{A/E}$
	1593 keV	2104 keV	2615 keV	1621 keV	2004-2074 keV	
	[%]	[%]	[%]	[%]	[%]	[%]
GD32A	90.0(9)	12.0(10)	16.8(7)	17.8(12)	43.4(4)	1.32(1)
GD32B	90.0(13)	5.6(8)	8.0(7)	10.0(9)	32.3(6)	0.76(1)
GD32C	90.0(20)	8.1(12)	13.8(7)	11.6(16)	42.4(7)	1.82(1)
GD32D-I	90.0(3)	11.8(16)	17.4(9)	18.6(17)	43.9(6)	1.42(3)
GD32D-II	90.0(9)	6.1(7)	8.7(5)	9.0(9)	39.6(4)	0.32(1)
GD35A	90.0(3)	9.0(10)	13.3(7)	12.9(12)	36.9(5)	3.23(3)
GD35B	90.0(12)	5.0(8)	6.4(6)	9.9(11)	32.3(6)	0.30(1)
GD35C	90.0(6)	11.2(9)	16.3(6)	16.0(10)	41.7(4)	2.95(3)
GD61A	90.0(7)	7.0(5)	9.9(4)	11.5(9)	39.8(4)	0.94(1)
GD61B	90.0(8)	8.5(8)	13.7(5)	13.5(9)	45.4(3)	1.22(1)
GD61C	90.0(3)	7.4(4)	10.2(3)	12.4(7)	41.4(4)	0.49(2)
GD76B	90.0(10)	21.2(19)	34.7(11)	21.9(27)	48.6(5)	1.92(6)
GD76C	90.0(8)	5.6(6)	7.0(6)	8.9(10)	37.1(7)	0.40(3)
GD79B	90.0(10)	11.9(7)	16.4(4)	17.7(7)	48.4(3)	1.77(2)
GD79C	90.0(6)	7.4(5)	12.8(3)	13.0(6)	44.8(2)	2.22(2)
GD89A	90.0(9)	10.6(8)	17.0(5)	16.5(10)	48.5(3)	1.25(1)
GD89B	90.0(11)	7.5(6)	12.4(4)	12.3(8)	43.8(3)	1.51(4)
GD89C	90.0(9)	9.1(6)	13.2(4)	14.3(10)	46.3(3)	0.51(1)
GD89D	90.0(5)	8.5(9)	14.8(9)	16.4(21)	47.4(6)	1.38(2)
GD91A	90.0(11)	8.0(8)	11.9(5)	11.9(7)	43.3(4)	0.94(1)
GD91B	90.0(8)	8.5(7)	12.2(4)	13.3(9)	43.8(3)	1.09(2)
GD91C	90.0(11)	11.6(6)	17.4(4)	17.8(8)	49.5(2)	2.67(1)
GD91D	90.0(4)	7.0(5)	10.6(3)	11.7(7)	42.4(3)	0.47(1)
GD00A	90.0(6)	10.8(5)	16.9(4)	16.6(9)	49.7(2)	1.79(1)
GD00B	90.0(9)	8.9(7)	12.5(6)	11.0(12)	44.1(5)	1.29(2)
GD00C	90.0(8)	6.4(6)	10.0(4)	10.3(6)	41.0(4)	0.56(1)
GD00D	90.0(8)	6.0(6)	9.1(4)	9.9(7)	38.9(4)	0.60(1)
GD02A	90.0(5)	11.8(7)	18.6(4)	18.4(8)	49.9(2)	3.58(1)
GD02B	90.0(7)	7.4(8)	13.8(6)	11.4(9)	45.8(4)	0.68(3)
GD02C	90.0(9)	7.6(6)	11.2(4)	11.9(10)	42.3(4)	1.96(3)
GD02D	90.0(7)	15.0(17)	27.6(11)	23.2(2.5)	56.1(8)	2.93(12)

*Gamma-ray background rejection using the* ‘*A* / *E* *ratio method’:*

*(a) Width of DEP* *A* / *E* *ratio distributions:* The $^{228}$Th-based ‘*A* / *E* ratio method’ requires a detailed examination of the *A* / *E* ratio distribution from DEP events to define the SSE/MSE cut. An example is shown in Fig. 14. Especially the width $b_{A/E}$($^{228}$Th) of this distribution is expected to be important: the narrower it is, the better the SSE/MSE separation. The calculated $b_{A/E}$($^{228}$Th) values are summarized in Table 10. They lie in the range between 0.32 % and 3.58 %, with an average of 1.42 % and a SD of 0.90 %. Looking at the individual detectors, the following situation appears:

- 11 detectors: $$b_{A/E}$$($$^{228}$$Th)<1 %.
- 13 detectors: 1$$\le b_{A/E}$$($$^{228}$$Th)<2 %.
- 6 detectors: $$b_{A/E}$$($$^{228}$$Th)$$\ge $$2 %.

The origin(s) of the unexpected broader $b_{A/E}$($^{228}$Th) values and partly multiple-structured DEP *A* / *E* ratio distributions in the latter two cases were thoroughly investigated. A deterioration of the electronics read-out, but also the potential impact of detector intrinsic properties such as the net impurity concentrations, were excluded. Finally, a series of hints from detector reprocessing and thermal cycles of single detectors pointed towards a common origin of this ‘*A* / *E* ratio anomaly’: negatively charged compounds/particulates accumulated inside the groove during diode production. In ADL3- and siggen-based simulations we tried to reproduce such DEP *A* / *E* ratio distributions. One example is shown in Fig. 14. By simulating ${25}\times$10$^{10}$/cm$^2$ negative charges (4 $\upmu$C) deposited on the groove surface of GD89C, siggen succeeds to reproduce the measurement very well. However, it is not yet understood how such a large amount of charge was able to stick to the small groove surface during manufacturing.

**Fig. 15:**
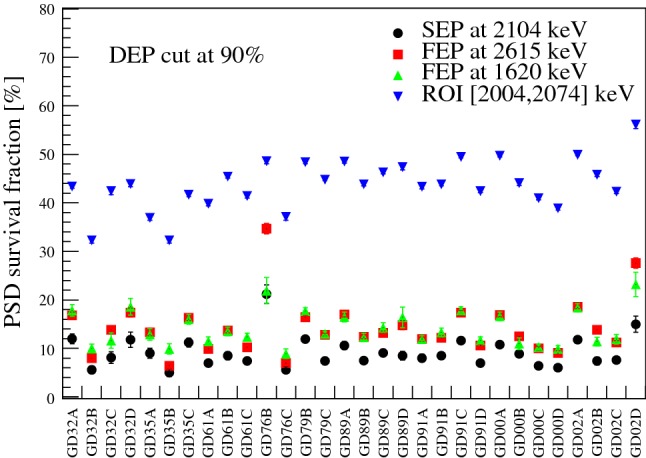
Illustration of the PSD survival fractions of different $^{228}$Th-induced $\gamma$-ray background in all 30 Gerda Phase II BEGe detectors

*(b) PSD survival fractions* This paragraph focuses on the capability of the Gerda Phase II BEGe detectors to discriminate SSE from MSE generated from $\gamma$-ray background sources. The PSD survival fractions of several FEPs and Compton continua were deduced with 90 % of the signal-like DEP events being kept. The obtained values are plotted in Fig. 15 and reported in Table 10 with their statistical and systematical uncertainties [55]. The PSD survival fractions of events in the SEP, 2615  keV and 1621  keV FEPs, and in the ROI around $Q_{\beta \beta }$($^{76}$Ge), lie in the range of [5,21] %, [6,35] %, [9,23] %, and [32,56] %, respectively. The detector with the best performance is GD35B. The detectors with the worst performance are GD76B and GD02D. By excluding these two problematic detectors, the relatively large ranges of PSD survival fractions shrink to [5,12] %, [6,19] %, [9,19] % and [32,48] %, respectively.

*(c) Correlations of the PSD survival fractions with other parameters* The last paragraph compares the obtained PSD survival fractions with other PS quantities and detector parameters.

**Fig. 16:**
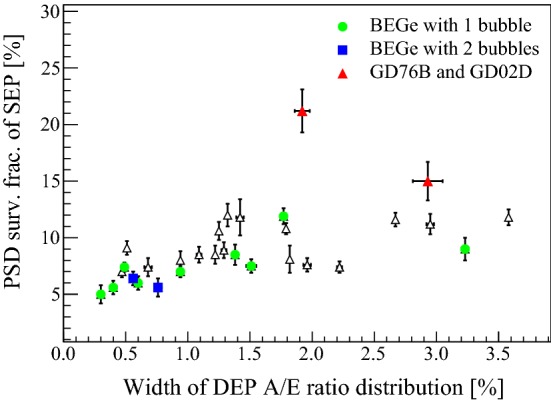
PSD survival fraction of $^{228}$Th SEP vs. the DEP *A* / *E* resolution for all 30 Gerda Phase II BEGe detectors operated under vacuum conditions. Open symbols are used like in Fig. 6

First, they were compared with the width of the DEP *A* / *E* ratio distributions $b_{A/E}$ ($^{228}$Th). The corresponding scatter plot with the $^{228}$Th SEP survival fractions is depicted in Fig. 16. As one can see, a dependence exists especially for $b_{A/E}<$1 %, while at larger values this trend is less pronounced. The situation is very similar for the FEPs at 1621  keV and 2615  keV as well as for the energy interval [2004,2074]  keV. A similar trend is found for *A* / *E* values of single detectors before and after they were reprocessed or underwent thermal cycles. This can be seen in Table 10 for detector GD32D, which was reprocessed and had an improved pulse shape performance afterwards. It is observed, that at least for very low $b_{A/E}$($^{228}$Th) values the background rejection is more effective. The reason is that a broader *A* / *E* distribution for DEP events results in a low *A* / *E* ratio cut position and hence more MSE events are accepted.

A second study investigated whether there are correlations between $b_{A/E}$($^{228}$Th) and other detector parameters, which would permit recursively a fast diagnosis about the expected background rejection capability of a detector. Only one small correlation seems to exist: detectors with larger height/mass ratios prefer to populate the band with better PSD survival fractions. Hence, it seems that smaller net impurity concentration is not only beneficial for obtaining thicker and thus more massive diodes (and thus less channels in a low background experiment), but improve also the background rejection capability, while the energy resolution deterioration (cf. Fig. 7) will be minimal.

## Summary and conclusions

For Phase II of the Gerda experiment, we have procured 30 new ^76^Ge enriched Broad Energy Ge (BEGe) detectors by the company Canberra. Prior to their integration at the experimental site, the detectors have been thoroughly tested in vacuum cryostats. This characterization campaign has led to a very detailed and extensive survey of high purity Ge (HPGe) detectors of the same design. These studies have allowed to search for correlations between different parameters and to test electric field calculations based on the ADL3 and siggen codes. The most important experimental findings have been reported.

First, the characterization tests confirmed the excellent energy resolution of the new detectors, with an average FWHM = (1.73 ± 0.07)  keV at the reference 1333  keV $\gamma$ line. The obtained energy resolutions do not only represent an improvement compared to the former semi-coaxial design, but are in general the best values obtained by a detector technology employed in 0$\nu \beta \beta$ search. The energy dependence of the energy resolution was also investigated. The related but not well known Fano factor was estimated to be (0.079 ± 0.006). This is in agreement with recent results.

Second, a careful examination of the full depletion voltage of the detectors via high voltage scans allowed us to revise the values recommended by the manufacturer. The new values turned out to be on average 600 V lower than the recommended ones. This knowledge is used in Gerda Phase II to prevent the development of prohibitive high leakage currents in a few delicate channels. A correlation between full depletion voltage, net impurity concentration and diode dimensions could be established for the BEGe design. Moreover, the high voltage scans revealed that around 40 % of the new detectors are affected by the ‘bubble depletion’ effect. In most of these cases a single ‘bubble’ was observed, in two detectors even two independent ‘bubbles’ for the first time. In the simulation the ‘bubble effect’ spreads over several 100 V and the data (*PP* in Fig. 4) also showed a deviation for a larger interval. Thus, the successful reproduction of them by simulations is a unique validation test for the field calculation codes.

Third, a large effort was made to determine precisely the full charge collection depth (FCCD) and active volume (AV) of the detectors. The measurements were carried out with surface sensitive $^{241}$Am and $^{133}$Ba sources as well as with bulk sensitive $^{60}$Co $\gamma$ probes. Compared to $^{60}$Co, the results based on the first two sources turned out to have the smallest total uncertainties. Thus, these results were combined, then corrected for ageing effects caused by 3 years of storage at room temperature. The active mass of the 29 well working BEGe detectors amounts to ($17.13^{+0.32}_{-0.29}\text {(uncorr)}^{+0.12}_{-0.06}\text {(corr)})\,\text {kg}$. Even though 34 systematic effects were considered, it remained unclear why the mean FCCD values from the higher energetic $^{60}$Co source are systematically larger. One of few remaining explanations might be related to the charge cloud size model used in the simulation code. If the description is correct, then the FCCD/AV would be an energy-dependent quantity. However, if the models are incomplete, the discrepancy would be an artifact of the simulation.

Fourth, the pulse shape behavior of the BEGe detectors was investigated. To begin, fine-grained scans using collimated $^{241}$Am sources allowed to test the pulse shape response of events generated close to the detector surface. The scan data allowed to visualize the crystal lattice orientation due to the expected hole drift anisotropy, and electric field calculations were able to reproduce this result. Then, non-collimated $^{228}$Th source tests allowed to define the background rejection capability of $\gamma$-induced radiation from signal-like events. While keeping 90 % of the signal-like proxies, $\gamma$ lines were suppressed on average at (86–91) % level and the Compton-continuum events around the ROI by 56 %. This suppression is better than for the former semi-coaxial Ge detector design, but is slightly deteriorated compared to the prototype BEGe detectors. Some detectors showed good PSD performance and small width of the *A* / *E* parameter for DEP events similarly as we observed for the prototype BEGe detectors. Others have a much poorer performance which might be related to surface charges in the groove which is corroborated by tests as well as electric field calculations.

The 30 BEGe detectors are deployed in the Gerda experiment since more than 3 years. Their energy resolution and other pulse shape parameters show a good stability over the whole data taking period. Due to the increased noise level in the Gerda cryostat compared to vacuum cryostats, their PSD performance is slightly degraded. The suppression of FEPs, SEP and Compton continuum events in $^{228}$Th calibrations using the *A* / *E* ratio method is on average 84 %, 88 %, and 53 %, respectively, while keeping 90 % of the DEP events. This helped Gerda to reach the lowest background level in 0$\nu \beta \beta$ experiments.

To summarize, the performed BEGe measurement campaign offered a unique possibility to collect a large variety of results, out of those several were incorporated in the standard Gerda Phase II data collection and analysis procedure. It is emphasized, that the improved knowledge about the detector properties is essential for reduced systematic uncertainties in Gerda, e.g. the active mass or cut efficiencies. In addition, the developed and improved electric field calculations advanced to valuable tools to interpret observed phenomena in HPGe detectors. The combination of dedicated measurements and proper simulation codes has not only become useful for Gerda, but will also be important for future HPGe-based experiments such as Legend [56], that will face an even larger number of detectors.

## Data Availability

This manuscript has no associated data or the data will not be deposited. [Authors’ comment: Data generated during this analysis and shown in all figures and Tables 7 and 10 are available as png, pdf and tex files from the Gerda repository at Zenodo (10.5281/zenodo.3516250). For further information contact the Gerda Collaboration (gerda-eb@mpi-hd.mpg.de).]
